# The Ordinal Effects of Ostracism: A Meta-Analysis of 120 Cyberball Studies

**DOI:** 10.1371/journal.pone.0127002

**Published:** 2015-05-29

**Authors:** Chris H. J. Hartgerink, Ilja van Beest, Jelte M. Wicherts, Kipling D. Williams

**Affiliations:** 1 Department of Methodology and Statistics, Tilburg University, Tilburg, the Netherlands; 2 Department of Social Psychology, Tilburg University, Tilburg, the Netherlands; 3 Department of Psychology, Purdue University, West Lafayette, Indiana, United States of America; University of Groningen, NETHERLANDS

## Abstract

We examined 120 Cyberball studies (N = 11,869) to determine the effect size of ostracism and conditions under which the effect may be reversed, eliminated, or small. Our analyses showed that (1) the average ostracism effect is large (d > |1.4|) and (2) generalizes across structural aspects (number of players, ostracism duration, number of tosses, type of needs scale), sampling aspects (gender, age, country), and types of dependent measure (interpersonal, intrapersonal, fundamental needs). Further, we test Williams’s (2009) proposition that the immediate impact of ostracism is resistant to moderation, but that moderation is more likely to be observed in delayed measures. Our findings suggest that (3) both first and last measures are susceptible to moderation and (4) time passed since being ostracized does not predict effect sizes of the last measure. Thus, support for this proposition is tenuous and we suggest modifications to the temporal need-threat model of ostracism.

## Introduction

**Fig 1:**
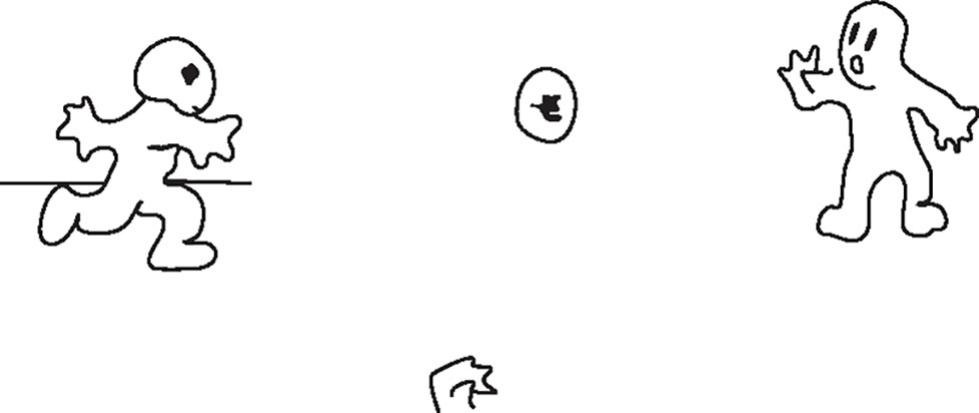
Cyberball game screenshot.

Cyberball [1] is a virtual ball-tossing game that is used to manipulate the degree of social inclusion or ostracism in social psychological experiments. In this game the participant supposedly plays with two (or more) other participants, who are in fact part of the computer program. The program varies the degree to which the participant is passed the ball (see Fig 1 for a still from the game). Ostracized players are not passed the ball after two initial tosses and thus obtain fewer ball tosses than the other players. Included players are repeatedly passed the ball and obtain an equal number of ball tosses as the other players. Our literature search showed that at least 200 published papers involved the use of the Cyberball paradigm to study ostracism and that over 19,500 participants have played the game thus far. In this paper we provide a meta-analysis of these studies. Our aim was to gauge the typical effect size of being ostracized in the Cyberball game and to see whether this effect is moderated by cross-cutting variables that were hypothesized to reduce/enhance the psychological impact of ostracism, structural aspects that are inherent in Cyberball (e.g., number of players, number of ball tosses), sampling aspects of the studies (e.g., gender composition), the type of dependent variables used (e.g., intrapersonal measures such as need satisfaction or interpersonal measures such as pro- or antisocial behavior), and the ordinal time point of the variable assessment (i.e., first or last).

### Historical background

Cyberball was introduced in 2000 as a means to study ostracism, that is: being excluded and ignored [1]. This focus of Cyberball on ostracism sets it apart from other paradigms that are tailored to study rejection, such as the future life rejection [2], the get-acquainted paradigm [3], and the autobiographical memory manipulation (i.e., remember a time when you were excluded [4]). The difference is that participants in Cyberball are not explicitly informed that they are excluded whereas in the other paradigms participants are provided a reason pertaining to why they are excluded. The Cyberball manipulation is a suitable method to study how people react to being ignored and excluded. Humans are social animals and care deeply about whether they are included or ostracized by others. Interestingly, ostracism is not only observed among loved ones, but on all levels of human organization. In fact, research suggests that most people are ignored and excluded at least once a day [3]. The social relevance is further evident in that ostracism not only affects the person who is ostracized (intrapersonal effects), but often also others (interpersonal effects). As a grim example, research on school shootings has suggested a direct link between ostracism and revenge. People who were ostracized may retaliate by murdering those responsible and sometimes even innocent bystanders [5]. The impact of ostracism is also evident in research findings using Cyberball. Through experimental work, it has been repeatedly shown that being ostracized has an effect on people—either on their psychological functioning (e.g., decreases in positive mood [6]) or on certain interpersonal behaviors (e.g., increases in social susceptibility or aggressive behaviors [7,8]). These experiments have highlighted the (mostly negative) impact of ostracism on fundamental needs (e.g., belonging [9]), mood, physiology (e.g., body temperature [10]), and various other constructs, including those measured with behavioral measures (e.g., conformity, compliance, aggression). In the current paper, we refer to the general effect of being ostracized compared to being included in Cyberball as the *ostracism effect*.

To capture how people respond to ostracism, Williams [11] proposed a temporal need-threat model of ostracism. Here he suggested three stages of the ostracism effect, namely: (1) a *reflexive* stage, (2) a *reflective* stage, and (3) a *resignation* stage. In the reflexive stage, the response to the ostracism sequence is immediate and occurs like a reflex. This initial response is theorized to be socially painful, threatening [9] and, following overdetection theory [12], should be easily detectable due to evolutionary over-sensitivity to cues of ostracism. Such a reflex would not take into account situational specifics and provides little room for coping. The reflex is proposed to affect primarily pain, fundamental needs, and emotional reactions (e.g., increased anger and sadness). The affected fundamental needs are belonging, self-esteem, control, and meaningful existence, typically measured by a need satisfaction scale [11]. According to Williams, measures of reflexive responses must occur during, or in the case of self-report measures, immediately following Cyberball (with the wording of the questions referring to how participants felt *during the game*). The *reflective* (or delayed) stage, which follows this immediate response, is subject to more rational thought and coping with the threats. Part of such coping is the necessity for fortification of the threatened fundamental needs. Coping can be measured both in terms of speed of recovery (higher levels of need satisfaction approaching the levels of included participants) and emotional, cognitive, and behavioral choices. The *resignation* stage occurs after prolonged ostracism, causing prolonged periods of pain and more fundamental need threat. If one is not able to fortify the fundamental needs, a prolonged ostracism sequence leads to feelings of helplessness, alienation, depression, and unworthiness. Because the resignation stage is hypothesized to occur only after prolonged and repeated exposure to ostracism (as in months or years), it is not feasible (and even unethical) to study resignation responses in laboratory experiments. Hence, in this paper we limit ourselves to studying the reflexive and reflective stages. For these stages, Williams asserts that moderation and variation of need satisfaction effects by individual differences and socially relevant factors (e.g., type of group from which one is excluded) will be less likely to occur for reflexive measures than for reflective measures.

### Goals of meta-analysis

A limited number of Cyberball experiments have been reviewed in other meta-analyses, but these meta-analyses had a different goal than the current meta-analysis. Previous meta-analyses focused on social rejection and not on ostracism [12,13], or focused only on a specific dependent variable (e.g., fMRI [14,15]). Importantly, none of these early meta-analyses were specifically set up to test Cyberball effects only. Consequently, we do not know how structural variables of Cyberball or sample characteristics affect the ostracism effect size. Moreover, none of these meta-analyses considered whether it matters if a specific variable is measured first or last. Thus, it remains unclear whether the ostracism effect size decreases or increases over time and whether immediate measures are more or less moderated by cross-cutting variables. The goal of our meta-analysis is to provide a comprehensive understanding of the Cyberball-induced inclusion versus ostracism effect size. Under what conditions, if any, is the effect size negative, zero, or especially small? Under what conditions is it especially large? To answer these questions we made several selection decisions (see also the Open Science Framework (OSF) where we preregistered all selections and hypotheses; https://osf.io/ht25n).

The first selection decision is that we considered only the first and the last dependent variable of all included studies. The reason for this selection was that it allowed us to gauge whether the effect sizes are affected by the time point at which the effects are measured. Another reason is that it served as a proxy to evaluate the hypothesis that immediate measures should be less affected by cross-cutting variables than more delayed measures.

A second decision is that we considered two different approaches to test whether first and last measures can be moderated by cross-cutting variables. This allowed us to test the robustness of our hypothesis across independent variables. The first approach to assess moderation was to conduct a meta-analysis on all studies that were explicitly designed to test whether being ostracized or included can be moderated by a cross-cutting factor. For this purpose we selected all the studies that included an experimentally manipulated moderator variable. Moreover, to meta-analyze the interaction term for first and last measure we followed the prediction of the authors in computing this interaction term. A potential limitation of our decision to follow the prediction of the authors is that the predictions may have been generated post-hoc on the basis of observed outcomes. For example, if authors used a 2 (ostracized vs included) x 2 (ingroup vs outgroup design) we followed the prediction of the authors to compute whether the interaction term denotes that ostracism is increased by an outgroup or decreased by an outgroup (specific calculations are reported in the methods section and formulae in the S6 File). Moreover, after computing the overall interaction terms we created dotplots in which we depicted the effect of ostracism across the two levels of the moderator and—perhaps more importantly—the effect of the moderator across the two levels of the ostracism manipulation. This was done to facilitate the interpretation of an interaction term and specifically to show whether cross-cutting variables have more impact on being included in Cyberball or more impact on being ostracized in Cyberball [16].

The second approach to test moderation was to assess if and how first and last measures are moderated by structural aspects of Cyberball (i.e., number of depicted Cyberball players, number of ball tosses used, duration of the game) and sample aspects (i.e., gender composition, country of origin, age). Note that the outcome of this analysis may thus also be used for future researchers to decide how to set up a game of Cyberball and whether effects generalize across age, gender, and country of origin. Because prior research has not explicitly manipulated structural aspects in controlled experiments we did not have a specific prediction whether increasing the number of players, ball tosses, and game duration would increase or diffuse the impact of ostracism. Given that the social aspects of an interdependent setting may be less evolutionary relevant for males than for females [17] and less relevant for older people than younger people [18], we explored whether an increase of male participants and mean age would decrease the ostracism effect. Moreover, considering that collectivism might influence the degree to which belonging is important [19], we used a categorization of continents (i.e., U.S., other western countries, Asian countries, and remaining countries) to explore whether a more collective orientation would be associated with larger ostracism effects. Finally, because some of the factors might be related (i.e., an increased number of ball tosses is likely to be associated with an increase in duration), we decided to use a regression approach in which all factors were entered simultaneously. A benefit of this approach is that it ensures that significant predictors have an impact above and beyond the impact of the other predictors.

The third decision is that we also checked the robustness of our findings across various dependent variables. More specifically, we coded whether the first and last measures belonged to the category of *interpersonal* variables assessing how ostracism impacts others or belonged to the category of *intrapersonal* variables assessing how ostracism impacts the self. Examples of interpersonal measures are donations to charity, helping behavior, money allocations in economic games, and aggression measures such as irritating sounds blasts or hot sauce allocation. These were initially coded into pro- and anti-social, but were collated into the category interpersonal due to small k the first measure (4 and 10, respectively) and last measure (8 and 6, respectively). Examples of intrapersonal measures are self-reported anger, self-esteem, control, and physiological measures such as body temperature or galvanic skin response. A benefit of classifying all variables into broad categories is that it increases the power of the meta-analysis since expanding the analysis to even more specific constructs would seriously limit the number of available studies. We made one exception and that is that we also ran tailored analyses on a subset of the intrapersonal measures that assessed *fundamental needs* (i.e., belonging, self-esteem, control, and meaningful existence). These fundamental needs measures included the typical need satisfaction measures that are especially designed for Cyberball [1,20,21] and conceptually related measures such as the Rosenberg Self-Esteem Scale. The reason why we did focus on this specific subset of intrapersonal variables is that the evidence supporting Williams’ temporal model is to a large extent based on studies using these specific dependent variables. In other words, these fundamental needs measures are particularly important for testing Williams’s [11] prediction concerning moderation of ostracism effects over time.

### Hypotheses

Following our preregistered report on OSF, we divided the hypotheses into two primary hypotheses and several secondary hypotheses. The two primary hypotheses were: is there an ordinal decrease of the ostracism effect across time of measurement? (Hypothesis 1) and is there an ordinal difference in the interaction effect across time of measurement (Hypothesis 2)? Secondary hypotheses regarded moderation of the ostracism effect by structural aspects of the studies, sampling aspects of the studies, and different types of dependent measures used. These hypotheses will be answered with random and mixed-effects meta-analytic models applied to all 120 studies that we were able to collate.

## Method

### Study inclusion criteria

First, we only considered Cyberball experiments that contained a factor that manipulated the number of virtual ball tosses obtained by the participants. For this ostracism factor we only considered the condition in which participants were ostracized by all other participants and the condition in which participants were equally included by all other players. Second, we only considered experiments that incorporated a between-subjects design with random assignment. Within-subject designs were excluded, because this would require the correlations between measures in primary studies and such correlations are often not reliably reported in the papers. Moreover, most within-subjects designs regard high-dimensional neurophysiological measurements such as fMRI that are beyond the scope of this meta-analysis [14,15]. Third, we checked whether the experiments contained other factors besides the ostracism factor. If the experiment contained more than two additional factors we collapsed effects sizes across the factor that authors expressed least interest in. Moreover, continuous variables that were dichotomized into factorial levels were also collapsed due to the many problems dichotomization can cause (e.g., underestimation of effect size, spurious effects [22,23]; four cases). Fourth, for the dependent measures the criterion was that they were (expected to be) affected by the ostracism manipulation. We considered the measures that immediately followed the manipulation (first measure) and the measure at the end of the study (last measure), while excluding manipulation checks in this assessment.

Reasons for these inclusion criteria are threefold: (1) Most Cyberball experiments take place in such a format, making it an encompassing criterion for the purposes of this meta-analysis. (2) The choice to limit the meta-analysis to between-subject designs rendered computational aspects more feasible based on reported statistics in papers. (3) The criteria maximize experimental rigor as they minimize the need for subjective quality assessment of the primary studies. Indeed, clear inclusion criteria decrease variability due to design characteristics, which increases power for moderator analyses [24].

### Literature search

To have a comprehensive meta-analysis of Cyberball studies, we used seven search strategies in the period of November 2012 through April 2013. These search strategies included database searches, a call for data, cross-reference with Kip Williams’s online list of Cyberball studies, Google Scholar alerts, citation records, Society for Personality and Social Psychology (SPSP) conference abstracts, and personal communications.

The databases searched included Web of Knowledge, PubMed, ScienceDirect, and Worldcat using all sources from the Tilburg University library. The first three cover only published articles, whereas Worldcat also covers books and dissertations as well as the PsycINFO database. All these databases were searched with the keywords *cyberball*, *ball-tossing* and ball AND ostraci*. Web of Knowledge was the first database searched. For this database, an additional search term (i.e., ball AND exclu*) was used, but this additional search term yielded zero relevant hits that were not a result of the other searches and was dropped. Across all these searches, results included 1927 potentially relevant studies of which a total of 109 were deemed relevant and saved for coding. Within Web of Knowledge, we looked through all citation records of the seminal papers by Williams et al. [1]; Williams and Jarvis [25]. These papers were cited 332 times (as of 5^th^ of November, 2012), of which 43 papers were saved for coding. The entire literature search provided 2259 potentially relevant studies (including possible duplicates across searches), of which 152 were selected to be included in the coding.

The call for data was put on the list servers or forums of SPSP, European Association of Social Psychology (EASP), and Social Psychology Network (SPN; all on 3rd of December, 2012). This resulted in 9 replies, yielding 3 useful studies.

Kip Williams keeps a list of Cyberball studies on his website. This list was used to check for extra articles that did not turn up in the initial searches on November 15^th^, 2012. It has been updated since, but the list that was used can be found on the Open Science Framework. The used list included 93 papers, of which 9 papers were included to be coded.

The final searches included Google Scholar alerts, SPSP conference abstracts, and personal communication. The Google Scholar alerts were used to keep up to date with new literature. These alerts notify a user when new search results for a search term occur and were used for *cyberball* and *ball-tossing*. This yielded 85 search results of which 25 were saved for coding. SPSP conference abstracts from 2006 through 2013 were searched for Cyberball studies. This led to personal communications with the authors of the conference abstracts, leading to additional studies. Pooled, the personal communication and the conference abstracts yielded 21 potentially relevant studies, of which 20 were saved for coding. The seminal paper by Williams et al. [1] was added separately.

**Fig 2:**
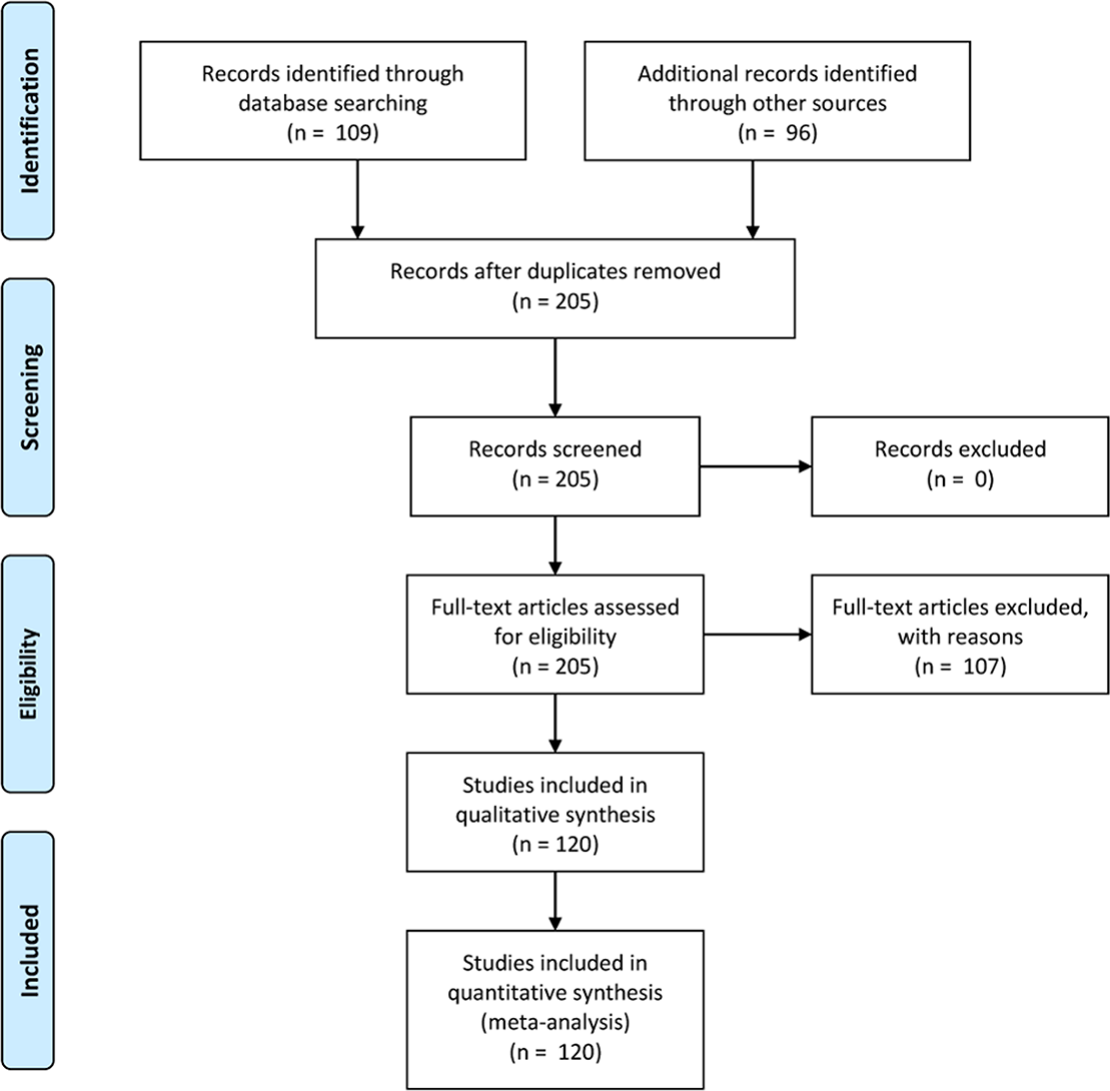
PRISMA flowchart of the current meta-analysis.

In sum, the literature search spanned 2468 potentially relevant studies, resulting in 205 that were saved for coding. During coding, papers were assessed to fit the inclusion criteria. Of the 205 papers, 107 papers were excluded for a variety of reasons. See also Fig 2. Several involved the use of a within-subjects design (52 papers). Some papers could not be accessed (5 papers) or could not be included because we did not receive the required data on request (7papers). Some were excluded for other reasons (43 papers), such as not involving new data (e.g., a dissertation study that was later published). All included papers were published between 2000 (after the introduction of Cyberball) and April 2013. This resulted in a final, fully coded sample of 98 papers containing 120 studies, with mean sample size 98.9 and median sample size 74. Oaten, Williams, Jones and Zadro [26] was applicable, but was excluded due to being an outlier with respect to effect size (*d*s > 15; see also Gerber and Wheeler, 2009; p. 473). There were a total of 11,869 Cyberball participants.

### Coding procedure

The first author coded all the studies and conducted all the analyses. The second author double-checked the coding of all 52 studies that entailed a full two-by-two design. Agreement between the first and second author was reached by discussion. We did not record these discussions and intercoder reliability cannot be assessed. The third author double-checked and reran the R code of all analyses. Finally, an extensive account of all coding decisions is publicly available via Open Science Framework on a paper-by-paper basis (see Footnote 2 for the direct link, S1 File also contains the data).

We first coded the structural aspects and sample aspects of all papers. The structural aspects of Cyberball that we coded were (1) number of players depicted in Cyberball, (2) total number of ball tosses used throughout the game, (3) total duration of the game in seconds. The sample aspects that we coded were (1) percentage of male participants, (2) average age of participants, and (3) country of origin.

We then coded the dependent variables that were relevant for the current meta-analysis by retrieving the means and standard deviations of the first and the last relevant measure of all papers. Importantly, to estimate the duration between the first and last measure we counted the number of questions that were assessed between the two measures. Specifically, following a longstanding practice in the freshman testing program of the University of Amsterdam [27] we estimated that participants would need 6 seconds on average to complete one question. Moreover, we included additional time if this was explicitly reported in the method section of the manuscript or when a measure would clearly deviate from 6 seconds to complete (e.g., tasks that measure endurance such as a grip strength task).

Both first and last measures were subsequently coded in the following general terms: (1) interpersonal, (2) intrapersonal, (3) fundamental needs, (4) model correspondence. Interpersonal measures were defined as measuring constructs that relate to (the self and) others (e.g., *how angry do you feel towards person X?*, donations to charity). Intrapersonal measures were defined as measuring constructs that relate only to the self (e.g., *how angry do you feel?*, physiological measures). Fundamental needs measures were those that measured self-esteem, belonging, control, meaningful existence, or a composite of these. Note that the fundamental needs are a refinement of the intrapersonal measures and that intrapersonal measures thus include the fundamental need measures. The model correspondence variable coded whether the first- and last measure fit the definition William’s ostracism model that a variable can indeed be classified as an immediate measure (i.e., during the game) and delayed measure (i.e., after the game/now), respectively.

The consequence of including many different kinds of dependent variables is that some measures are expected to increase as a function of ostracism (e.g., need threat) and others are expected to decrease (e.g., need satisfaction). To counteract computational problems (i.e., cancellation of effects) being caused by this bidirectionality of ostracism effects, we coded the direction of the ostracism effect for each specific measure, such that negative effect sizes depict negative psychological effects.

A similar argument can also be made about including multiple moderator variables in the analysis of interaction effects. In the 52 studies that included a moderator variable we thus needed to account for the expected direction of every moderator. If we had not done this, the interaction effects could cancel out, thereby leading to ambivalent results. To explain this, we present in Table 1 hypothetical data for the four different study designs that are possible when crossing direction of the effect and direction of the moderation. The relevant effect sizes should be corrected to attain comparable effect sizes across studies. Effect sizes for the simple ostracism effect (column wise) were corrected only for the type of measure. For instance, for panels (a) (involving, e.g., need threat) and (c) (involving, e.g., need satisfaction), the corrections entailed a multiplication with -1 or +1, respectively. Simple moderator effects (row wise comparisons) are interesting for understanding the effect of the moderator under either ostracism or inclusion. These simple moderator effects were corrected for both the type of measure *and* the expected moderation (i.e., exacerbation, -1, or minimization, +1). For example in panel (c), the 5 and 8 on the right are used to compute the *standard ostracism effect* (as in [1]), whereas the 3 and 8 in the left column represent an ostracism effect that is thought to be exacerbated. For example, in a given ostracism study with a two-by-two design, adolescents are expected to show stronger ostracism effects, compared to young adults [18]. The 5 and 8 would subsequently represent the scores for the young adults, whereas the 3 and 8 would represent the scores for the young adolescents. In panel (d) we depict a study in which the *moderated* column is thought to lead to a minimal ostracism effect, as could be expected when Cyberball is played with members of a despised out-group [28]. The margins (greyed out) denote the simple effects, which are after correction comparable across all panels (a) through (d), indicating that this correction did what we intended it to.

**Table 1:** Hypothetical data example of coding correction.

(a) Negative moderator, negative measure						(b) Positive moderator, negative measure					
		Moderated	Not-moderated/control	Raw	Correct			Moderated	Not-moderated/control	Raw	Correct
Ostracism factor	Ostracism	13	11	2	2	Ostracism factor	Ostracism	9	11	-2	2
	Inclusion	8	8	0	0		Inclusion	8	8	0	0
	Raw	5	3				Raw	1	3		
	Correct	-5	-3				Correct	-1	-3		
(c) Negative moderator, positive measure						(d) Positive moderator, positive measure					
		Moderated	Not-moderated/control	Raw	Correct			Moderated	Not-moderated/control	Raw	Correct
Ostracism factor	Ostracism	3	5	-2	2	Ostracism factor	Ostracism	7	5	2	2
	Inclusion	8	8	0	0		Inclusion	8	8	0	0
	Raw	-5	-3				Raw	-1	-3		
	Correct	-5	-3				Correct	-1	-3		

Raw denotes the simple effect in the hypothetical data before correction whereas correct denotes the simple effect after correction. Column wise effects are multiplied by the type of measure only, whereas row wise effects are multiplied by both the type of moderator and type of measure.

Finally, relevant information that was missing in the papers was requested from the authors via e-mail. In case of non-response, we sent three follow-up e-mails. All this communication was documented and can be found on the OSF page for this project. In case of non-response or non-willingness to send data, studies were either eliminated if the information was crucial (i.e., means and standard deviations of the measures per group), computed if possible (i.e., cell sizes), or assumed if deemed reasonable on the basis of additional information. For instance, when no information was given we considered the Cyberball manipulation characteristics to be similar to previous studies in the same paper or in earlier papers referred to in the paper (descriptions of all cases are described in the log file on the OSF).

### Statistical analyses

For the analyses, we used version 1.9–5 of the *metafor* package [29] in the R statistical environment [30].

#### Effect size metric

We used Hedges’s *g* version of the standardized mean differences as the effect size. Hedges’s *g* corrects for the slightly biased estimate given by Cohen’s *d* [31]. Standardized effects were calculated across the ostracism factor, where the 52 studies with a cross-cutting variable were included as a simple effect of ostracism within the non-moderated level. Standardized interaction effect were calculated by taking the standardized difference between the unstandardized main effects (see S6 File for the exact formulae used). These effects were computed for both the first and last dependent variable in each experiment. For example, in a 2 (ostracized vs. included) by 2 (moderator present vs. moderator absent) design with multiple measures, we calculated two simple ostracism effects (Hypothesis 1) and two interaction effects (Hypothesis 2). For ten studies, more factors/levels were used and a 2 by 2 was extracted.

#### Meta-analytic model

We used random- and mixed-effects models, because heterogeneity in the effect sizes is expected due to both the inclusion of different measures and additional unknown methodological and substantive factors. The meta-regression element in some of the analyses is the variable time as predictor of the ostracism effect. Analyses without this study-level predictor reduce to a random-effects model. We used Restricted Maximum Likelihood (REML) to estimate tau-squared (i.e., the residual variance), as recommended by Viechtbauer [32]. Note that when estimating a mixed- or random effects model, one does not estimate a single *true* effect, but rather the mean and variance of underlying effects [32].

#### Statistical sensitivity analyses

To test for robustness of the effects, we incorporated several statistical sensitivity analyses. We flagged possibly problematic outliers on the basis of studentized deleted residuals, Q-Q plots, and Cook’s distance values. Subsequently, we inspected the effect of these outliers on substantial results in statistical sensitivity analyses in which these outliers were excluded. Another statistical sensitivity analysis entailed fitting of the mixed-effects model with tau-squared fit at the upper bound value of the 95% confidence interval.

#### Funnel plot asymmetry

A funnel plot depicts each study’s effect size against its standard error [33]. Larger studies have smaller standard errors, and vice versa for smaller studies. Following from a theoretical fluctuation of the population effect size due to sampling variance, a funnel plot should be symmetrical around the estimated mean effect size. If there are no methodological or substantive reasons to expect a link between effect sizes and standard errors, funnel plot *asymmetry* can indicate publication bias (e.g., [34]). To test funnel plot asymmetry, we used Egger’s regression test [35] for mixed-effects models [36]. Due to dependency between the standardized effect size and the standard error, we also ran an alternative version of the Egger’s test that regresses on 1/N. These analyses yielded highly similar results. Egger’s regression test inspects whether the distribution of effect sizes is equal on both sides of the average effect, when accounting for true heterogeneity. Funnel plot asymmetry thus indicates bias in the estimated mean effect size and possibly publication bias.

## Results

In our reporting of the effect sizes, *d* indicates a main effect and Δ*d* indicates an interaction effect. Even though we used Hedges’s *g*, we maintained the notation of *d*, because *g* is only a minor correction to Cohen’s *d*. Statistical sensitivity analyses are only reported if they showed different effects (all statistical sensitivity analyses can be found on OSF).

### Primary analyses

The two primary hypotheses are tested in four meta-analyses, of which the study level effects are reported in Table 2. The table includes effect sizes used in the estimation of the average simple effect of ostracism on the first measure, the average simple effect on the last measure and the estimation of the average interaction effect on both the first and last measure.

**Table 2:** Effect sizes per study for the primary hypotheses.

First author	Year	*N*	*d* T1	(*SE*)	*d* T2	(*SE*)	Δ*d* T1	(*SE*)	Δ*d* T2	(*SE*)
Alvares	2010	74	-1.21	0.12	-0.10	0.10	-0.15	0.24	1.12	0.23
Ambrosini	2013	40	-1.69	0.13	-0.97	0.11	-	-	-	-
Aydin	2012	68	-0.95	0.13	-0.40	0.12	-1.19	0.24	0.72	0.23
Banki	2012	89	-1.87	0.07	-0.35	0.05	-	-	-	-
Bastian	2010	72	-2.75	0.11	-1.42	0.07	-	-	-	-
Bernstein	2012	24	-0.41	0.16	-	-	-	-	-	-
Bernstein	2012	25.50	-1.04	0.17	-	-	-	-	-	-
Bernstein	2010	73	-1.63	0.16	-1.63	0.16	-0.86	0.37	-1.11	0.40
Bernstein	2010	138	-2.67	0.10	-1.96	0.08	-0.53	0.22	-0.51	0.17
Bernstein	2012	67	-2.00	0.17	-0.99	0.13	-1.07	0.45	-0.80	0.30
Bernstein	2012	27	-1.39	0.17	-	-	-	-	-	-
Boyes	2009	89	-0.43	0.05	-0.80	0.05	-	-	-	-
Boyes	2009	87	-0.20	0.05	-0.84	0.05	-	-	-	-
Brochu	-	35	-2.51	0.20	-0.48	0.11	-	-	-	-
Brown	2009	52	-0.64	0.08	-	-	-	-	-	-
Carter	2008	143	-0.28	0.06	0.20	0.06	0.34	0.11	0.17	0.11
Carter-Sowell	2008	65	-2.86	0.12	-1.48	0.08	-	-	-	-
Carter-Sowell	2010	74	-1.60	0.14	-1.49	0.13	-1.23	0.33	-1.15	0.34
Carter-Sowell	2010	70.67	-2.09	0.17	-0.56	0.11	-0.65	0.39	-0.63	0.24
Chen	2012	60	-1.04	0.14	-	-	-1.35	0.27	-	-
Chen	2012	83	-1.32	0.11	-	-	-1.32	0.21	-	-
Chernyak	2010	76	-1.52	0.10	0.15	0.08	-	-	-	-
Chow	2008	75	-1.20	0.06	-1.31	0.06	-	-	-	-
Chrisp	2012	77	-0.70	0.06	-0.15	0.05	-	-	-	-
Coyne	2011	40	-0.56	0.10	-	-	-	-	-	-
De Waal-Andrews	2012	136	-3.55	0.16	-2.55	0.11	-1.29	0.24	-0.87	0.18
De Waal-Andrews	2012	112	-4.21	0.22	-2.17	0.11	-1.56	0.31	-1.20	0.18
DeBono	-	57	-1.07	0.15	-0.05	0.13	-1.55	0.29	-0.48	0.27
DeBono	-	81	-1.07	0.11	-0.10	0.09	-0.33	0.21	0.24	0.19
DeBono	-	83	-0.13	0.09	-	-	-0.75	0.19	-	-
Dietrich	2010	75	1.43	0.07	-	-	-	-	-	-
Duclos	2012	59	-0.63	0.07	-	-	-	-	-	-
Eisenberger	2006	48	-0.15	0.08	-1.24	0.10	-	-	-	-
Fayant	-	60	-2.04	0.20	-1.12	0.15	0.22	0.38	-0.44	0.28
Floor	2007	88	-1.92	0.13	-0.73	0.09	-0.21	0.28	-0.59	0.19
Gallardo-Pujol	2012	57	-1.18	0.16	-0.52	0.15	-1.17	0.31	0.11	0.29
Gan	2012	72	-0.54	0.03	-0.07	0.03	-0.62	0.06	0.02	0.06
Garczynski	2013	83	-1.51	0.19	0.39	0.15	-1.29	0.33	-0.01	0.29
Geniole	2011	74	0.19	0.06	-0.11	0.06	-	-	-	-
Gerber	-	38	-2.09	0.16	-	-	-	-	-	-
Gerber	-	89	-3.38	0.21	-	-	-	-	-	-
Gonsalkorale	2007	97	-1.31	0.14	0.26	0.12	0.49	0.30	1.31	0.25
Goodwin	2010	300	-1.81	0.04	-0.94	0.03	0.20	0.08	-0.43	0.07
Goodwin	2010	314	0.13	0.02	-0.09	0.02	0.35	0.06	-0.10	0.06
Greitemeyer	2012	56	-0.48	0.07	-0.23	0.07	-	-	-	-
Gruijters	-	113	-0.26	0.06	-1.07	0.07	-	-	-	-
Hackenbracht	2013	51	-1.92	0.11	-0.18	0.08	-	-	-	-
Hawes	2012	55	-2.16	0.23	0.69	0.15	0.00	0.38	-1.05	0.28
Hellmann	-	76	-1.21	0.12	0.19	0.10	-1.40	0.22	0.74	0.21
Hess	2010	162	-2.34	0.04	-0.87	0.03	-	-	-	-
Hess	2011	38	-0.64	0.11	-	-	-	-	-	-
Horn	-	68	-0.77	0.12	-0.99	0.13	-0.99	0.23	1.49	0.24
IJzerman	2012	86	-1.67	0.12	-	-	-1.07	0.22	-	-
Jamieson	2010	33	-1.56	0.15	-1.06	0.13	-	-	-	-
Jamieson	2010	68	-1.94	0.09	-1.47	0.07	-	-	-	-
Johnson	2010	104	-0.73	0.04	-0.79	0.04	-	-	-	-
Kassner	-	85	-1.72	0.13	-1.02	0.11	-0.87	0.31	-0.30	0.21
Kassner	2012	49	-2.11	0.12	-1.78	0.11	-	-	-	-
Kerr	2008	250	-1.66	0.02	-0.05	0.02	-	-	-	-
Kesting	2013	76	-0.28	0.05	-0.79	0.06	-	-	-	-
Knowles	2010	62	-0.38	0.12	-	-	-0.99	0.25	-	-
Knowles	2012	60	-0.60	0.07	-	-	-	-	-	-
Krijnen	2008	144	-4.74	0.11	-0.18	0.03	-	-	-	-
Krill	2008	119	-2.11	0.05	-0.57	0.03	-	-	-	-
Lakin	2008	36	-1.53	0.14	-0.51	0.11	-	-	-	-
Lau	2009	56	-2.50	0.23	-1.09	0.15	-0.06	0.58	1.36	0.46
Lustenberger	2010	71	-0.83	0.06	0.04	0.06	-	-	-	-
Lustenberger	2010	156	-0.70	0.03	-	-	-	-	-	-
MacDonald	2008	63	-0.15	0.06	-	-	-	-	-	-
McDonald	2012	270	-0.06	0.02	-2.40	0.03	-	-	-	-
Nordgren	2011	71	-0.74	0.06	-	-	-	-	-	-
Nordgren	2011	74	-0.80	0.06	-	-	-	-	-	-
Nordgren	2011	46	-2.24	0.14	-	-	-	-	-	-
Nordgren	2011	44.67	-0.55	0.09	-0.75	0.09	-	-	-	-
Nordgren	2011	58.67	-0.65	0.07	-	-	-	-	-	-
Oberleitner	2012	88	-2.36	0.08	0.42	0.05	-	-	-	-
O’Brien	2012	125	-0.58	0.03	-0.69	0.03	-	-	-	-
Peterson	2011	40	-0.89	0.11	-0.91	0.11	-	-	-	-
Pharo	2011	74	-1.33	0.13	-0.58	0.11	-1.01	0.30	-0.84	0.23
Plaisier	2012	149	-0.36	0.05	0.23	0.05	-0.40	0.11	-0.56	0.11
Ramirez	2009	121	-2.26	0.05	-1.02	0.04	-	-	-	-
Ren	2012	53	-2.18	0.12	-0.17	0.07	-	-	-	-
Renneberg	2011	60	-1.46	0.16	-1.30	0.15	0.47	0.29	0.51	0.29
Riva	2011	100	-2.10	0.13	-1.09	0.09	-	-	-	-
Ruggieri	-	91	-0.39	0.04	-0.57	0.05	-	-	-	-
Ruggieri	-	74	-0.06	0.13	-0.23	0.13	-0.31	0.24	-0.68	0.23
Sacco	2011	51	-2.40	0.13	-1.45	0.10	-	-	-	-
Sacco	2011	21	-2.28	0.29	-1.46	0.22	-	-	-	-
Sacco	2011	38	-1.74	0.14	-1.04	0.11	-	-	-	-
Salvy	2010	59	-1.45	0.08	-1.43	0.08	-	-	-	-
Salvy	2009	103	-1.48	0.05	-1.31	0.05	-	-	-	-
Schaafsma	2012	720	-1.42	0.02	-0.49	0.02	0.09	0.03	0.33	0.03
Segovia	2012	56	0.14	0.13	-	-	-1.89	0.32	-	-
Staebler	2011	68	-0.79	0.12	-0.05	0.12	0.50	0.23	0.42	0.23
Stillman	2009	121	-0.74	0.15	-1.13	0.16	0.57	0.22	-1.19	0.24
Stock	2011	155	-2.00	0.04	-0.13	0.03	-	-	-	-
Van Beest	2011	87	-0.94	0.10	-0.58	0.09	-0.40	0.24	-0.44	0.19
Van Beest	2011	183	-2.64	0.13	-0.50	0.07	-0.76	0.22	-0.11	0.13
Van Beest	2006	135	-1.29	0.07	-0.65	0.06	-0.10	0.14	-0.13	0.12
Van Beest	2006	111.33	-2.11	0.11	0.09	0.07	-0.09	0.22	-0.19	0.14
Van Beest	2012	125	-2.68	0.11	-1.24	0.07	0.06	0.35	-0.23	0.15
Van Beest	2012	85	-3.10	0.20	0.05	0.09	-0.28	0.44	0.07	0.18
Van Beest	2013	49	-3.97	0.24	-1.32	0.10	-	-	-	-
Van Beest	2013	91	-3.17	0.20	-0.48	0.09	0.75	0.56	0.53	0.18
Van Dijk	-	51	-1.50	0.10	-0.04	0.08	-	-	-	-
Webb	-	170	-0.91	0.05	-0.38	0.05	0.03	0.10	0.04	0.09
Weik	2010	65	0.16	0.12	-0.22	0.12	-0.43	0.24	0.66	0.24
Wesselmann	2009	82	-0.71	0.10	-2.03	0.14	-1.30	0.24	-0.20	0.28
Wesselmann	2012	91	-1.46	0.06	-	-	-	-	-	-
Williams	2002	390	-0.39	0.01	-2.35	0.02	-	-	-	-
Williams	2000	732	-0.79	0.01	-1.44	0.01	-	-	-	-
Williams	2000	111	-0.26	0.06	-1.01	0.07	-0.20	0.15	-0.98	0.15
Wirth	2009	159.33	-2.29	0.08	-0.76	0.05	0.05	0.17	0.46	0.11
Wirth	2010	76	-0.96	0.06	-1.64	0.07	-	-	-	-
Zadro	2004	62	-1.63	0.16	-0.19	0.12	-0.11	0.32	-1.12	0.28
Zadro	2004	77	-1.75	0.14	-0.33	0.10	-0.29	0.28	-0.70	0.21
Zadro	2006	56	-3.70	0.19	-0.87	0.08	-	-	-	-
Zhong	2008	52	-0.72	0.15	-	-	-	-	-	-
Zoller	2010	57	-0.24	0.07	-0.09	0.07	-	-	-	-
Zwolinski	2012	56	-2.01	0.11	-0.28	0.07	-	-	-	-

*d* T1 refers to ostracism effect on first measure; *d* T2 refers to ostracism effect on last measure; Δ*d* represent interactions. Multiple rows for the same first author and year is possible due to multiple studies across papers. Non-integer *N*s arise from division of full sample *N* for included conditions, appropriate due to random assignment (e.g., two conditions out of 3, when sample is 56: (56 / 3) × 2 = 37.333). S2 File gives the full reference list of the papers in this table.

#### Simple ostracism effect (Hypothesis 1)

In a random-effects model on the main effect of ostracism (*k* = 120), residual heterogeneity was significant, *Q* (119) = 1395, *p* < .001, *I* ^*2*^ = 92.99% and estimated at τ^2^ = 0.90, 95% CI [0.70, 1.24]. The heterogeneity measure τ^2^ includes both the estimated proportion of explained variance at the study level and unexplained variance in the distribution of underlying effect sizes (i.e., τ_res_^2^). The analysis yielded an estimated average effect of *d* = -1.36, p < .001, 95% CI [-1.54, -1.18]. A random-effects version of the Egger’s test [36] indicated funnel plot asymmetry, *Z* = -6.14, *p* < .001. Due to the size of the average effect, hence large power to acquire significant outcomes in primary studies, we do not suspect publication bias to explain this asymmetry. In other words, immediately after being ostracized, the average ostracism effect is estimated at -1.36 standard deviation units, which entails a large effect [37].

Next, we fitted a mixed-effects regression model for the ostracism effect on the last measure (*k* = 95), including estimated time in seconds since completing the Cyberball game as predictor. Residual heterogeneity was significant, *Q*_*E*_ (93) = 803, *p* < .001 and estimated at τ_res_^2^ = 0.38, 95% CI [0.27, 0.54]. The intercept was estimated at *d*_*intercept*_ = -0.76, *p* < .001, 95% CI [-0.91, -0.61]. Moreover, the estimated time in seconds between exclusion in Cyberball and the moment at which the last measure was taken failed to moderate the average effect, *b* = 0.0069, *p* = .187, 95% CI [-0.0034, 0.0172]. However, we have to take into consideration the low power of the moderation analyses due to the large (residual) heterogeneity in effect sizes [24]. A regression test for mixed-effects model with moderator (i.e., including both the time and *SE* as predictor) showed no funnel plot asymmetry, *Z* = -0.72, *p* = .474. In short, long after ostracism has occurred (*M*_*time*_ = 4.85 minutes), ostracized participants on average scored around -0.73 standard deviation units lower when compared with included participants, an effect that does not appear to be moderated further by time passed since the ostracism occurrence.

Thus, results show a clear effect of ostracism on both the first and last measures, of which the latter is *not* predicted by our operationalization of time. The ostracism effect over time can also be inspected via confidence intervals. Comparing the 95% confidence intervals for the average ostracism effect on the first measure (i.e., [-1.54, -1.18]) and on the last measure (i.e., [-0.86, -0.59]) showed no overlap. Although the difference in average effect sizes between first and last measure cannot be formally tested (because of a lack of information on the correlation between measures in the primary studies), the mean difference is sizeable and CIs confirms our prediction that the average ostracism effect is smaller for the last measure. In fact, given the expected positive correlation between effects for first and last measures, the comparison of CIs is likely to be conservative [38]. Additionally, we noted that estimated residual heterogeneity was larger on the first- than on the last measure. We conclude that the average ostracism effects decreases from the first- to last measures and that study-level effects are more similar on the last measure.

#### Moderation of ostracism (Hypothesis 2)

To test moderation of the ostracism effect, we selected the factorial experiments that manipulated ostracism and another independent variable in between-subjects designs. A random-effects model on the interaction effect (Δ*d*) on the first measure (*k* = 52) showed heterogeneity in underlying effects, *Q* (51) = 103.24, *p* < .001, *I* ^*2*^ = 50.60% and an estimated τ^2^ = 0.19, 95% CI [0.07, 0.41]. The average interaction effect equaled Δ*d* = -0.46, *p* < .001, 95% CI [-0.64, -0.28], indicating a change in the ostracism effect due to the moderator level and vice versa (i.e., moderation of the ostracism effect). There was indication of funnel plot asymmetry in this analysis, *Z* = -2.43, *p* = .015. Thus, the data indicate that, across the board, the ostracism effect *can* be moderated on the first measure following the ostracism sequence, but it is possible that publication bias may have affected the interaction estimates.

On the last measure (*k* = 46), the mixed-effects model (with estimated time as predictor) for the interaction effect again showed residual heterogeneity, *Q*_*E*_ (44) = 100.82, *p* < .001 and estimated τ_res_^2^ = 0.21, 95% CI [0.10, 0.55]. The intercept of the interaction effect was estimated at Δ*d*_*intercept*_ = -0.20, *p* = .052, 95% CI [-0.402, 0.002] and no significant moderation of time was found, *b* = 0.011, *p* = .159, 95% CI [-0.0043, 0.0264]. The regression test with the time and SE as predictors showed no funnel plot asymmetry, *Z* = -0.68, *p* = .495. These results indicate that moderation of the average ostracism effect is *not* found at a later time point in the included studies and time itself does not moderate the computed interaction effects. However, statistical sensitivity analyses showed that this interaction *was* significant when we removed three outliers based on studentized residuals, Δ*d*_*intercept*_ = -0.32, *p* = .029, 95% CI [-0.60, -0.03], whereas the regression coefficient time continued to be non-significant, *b* = 0.0002, *p* = .207, 95% CI [-0.0001, 0.0006]. On the last measure, this indicates that the non-significant interaction effect is sensitive to outliers in the data.

To see whether the interaction effects changed from the first to the last measure, we again compared confidence intervals. On the first measure, the 95% CI was [-0.64, -0.28] whereas for the last measure, the 95% CI was [-0.32, 0.05]. Considering the overlap of these CIs, one needs to be careful to interpret this as a reduction in the moderation across the measures examined. It is clear, however, that the average effect size of the interaction does not increase from first to last measure.

### Secondary analyses

In addition to the simple effects over all studies, we analyzed subsets of studies that differ in type of dependent measure to study robustness of the effects. We also inspected whether sample composition, scale composition, and Cyberball specifics could predict the estimated effect size. Finally, we selected a homogeneous subset of studies to come to grips with the relatively large heterogeneity of simple main effects found for the primary hypotheses.

#### Measures

**Fig 3:**
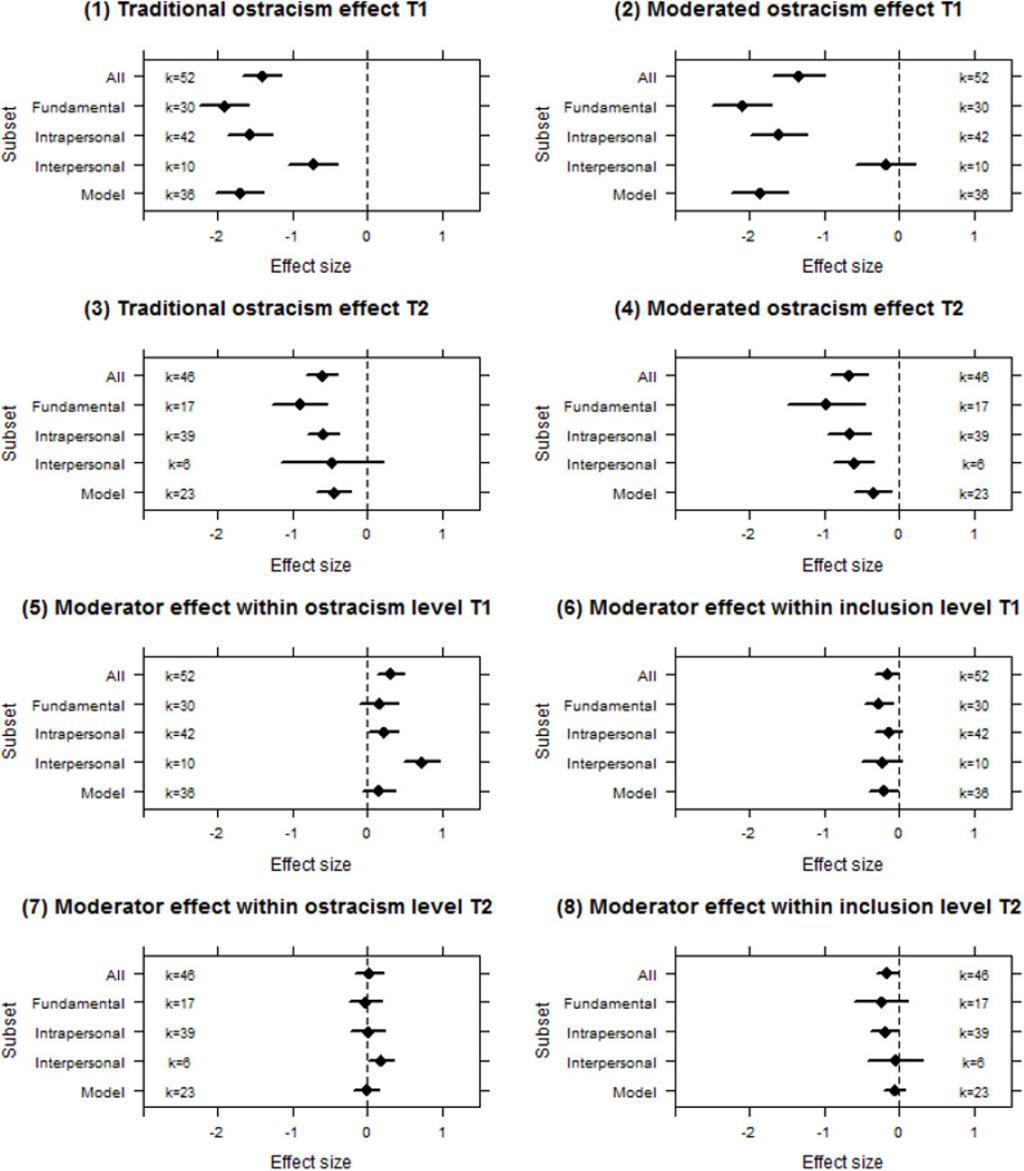
Dotplots of the average estimated simple effects with 95% confidence intervals. T1 represents first measure and T2 represents last measure. These effects are across the same subset. Traditional ostracism effect refers to the between-subjects effect of being ostracized with *no* moderator present, whereas moderated ostracism effect refers to being ostracized *with* a moderator present. Vice versa, moderator effect within ostracism/inclusion level refers to the between-subjects effect of the moderator factor, within the ostracized/inclusion conditions. The subset labeled “All” contains all measures. The subset labeled “Fundamental” contains only fundamental need measures. The subset labeled “Intrapersonal” contains all intrapersonal measures. The subset labeled “Interpersonal” contains all interpersonal measures. The subset labeled “Model” contains those where first measures is immediate and last measure is delayed. See S4 File.

To inspect the robustness of the estimates of the first and last measure, we studied simple effects across several subsets of measures. These subsets encompassed interpersonal measures (i.e., measures that relate to others or the self in the context of others), intrapersonal measures (i.e., measures that relate only to the self), fundamental needs (single- and composite needs), and measures that were coded by the first two authors as fitting the description of being immediate or delayed (i.e., questions related to during- or after the game, respectively; shown in Fig 3 as *model*). We ran the analyses for the different measures for the two time points separately (i.e., first and last measure).

The different panels in Fig 3 show the results for the different simple effects per subset and overall; Table 3 summarizes the estimated interaction effects. A comparison of the results within each panel shows whether the overall results are robust and representative of all subsets, or whether there are nuances per type of measure. The main differences are notable in panels (1), (2), and (5). The first and second panels indicate that the effect of ostracism is weaker for interpersonal measures, compared to all intrapersonal measures (including fundamental needs). This indicates that in a similar factorial design, interpersonal measures show weaker effects than intrapersonal measures. Panel 5 indicates that the moderation of interpersonal measures is stronger compared to the other subsets. This suggests that interpersonal measures are more subject to moderation, whereas the effects of ostracism on interpersonal measures are smaller initially. Additionally, for the specific subset of fundamental needs, we noted that the point estimated interactions (Table 3) follow the pattern predicted by the need-threat model [11]: the first measures are moderated less strongly than the last measures.

**Table 3:** Interaction effect per subset.

		*k*	Estimate	(*SE*)	*Z*-value	*p*-value	95% CI Lowerbound	95% CI Upperbound
Overall	T1	52	-0.46	0.09	-5.08	< .001	-0.64	-0.28
	T2	46	-0.19	0.11	-1.82	.069	-0.40	0.02
Fundamental	T1	30	-0.39	0.12	-3.42	< .001	-0.62	-0.17
	T2	17	-0.77	0.25	-3.05	.002	-1.27	-0.28
Intrapersonal	T1	42	-0.31	0.09	-3.38	< .001	-0.49	-0.13
	T2	39	-0.21	0.11	-1.87	.062	-0.44	0.01
Interpersonal	T1	10	-1.03	0.18	-5.69	<.0001	-1.38	-0.67
	T1_listwise_	6	-0.36	0.22	-1.63	.104	-0.79	0.07
	T2	6	0.63	0.62	1.02	.309	-0.58	1.84
Model	T1	36	-0.29	0.10	-2.99	.003	-0.48	-0.10
	T2	23	0.01	0.17	0.08	.938	-0.31	0.34

The subset labeled “All” contains all measures. The subset labeled “Fundamental” contains only fundamental need measures. The subset labeled “Intrapersonal” contains all intrapersonal measures. The subset labeled “Interpersonal” contains all interpersonal measures. The subset labeled “Model” contains those where first measures is immediate and last measure is delayed. See S4 File. Listwise deletion ensures that estimates are made on full rows in the data. Listwise deletion was applied in all the subsets, which only altered results for interpersonal measures.

Because fundamental needs showed effects in the theorized direction, we explored this further by overlapping the subset of fundamental need measures with the model definition of immediate and delayed (i.e., whether the measures related to feelings during or after the Cyberball game). Estimated interactions for this selection were Δ*d* = -0.37, 95% CI [-0.60, -0,14] (*k* = 29) and Δ*d* = -0.13, 95% CI [-0.53, 0.27] (*k* = 8) for the first and last measure, respectively. So in this particular subset of studies that use immediate or delayed fundamental needs measures, results are not in line with Williams’s [11] prediction. The reported fundamental need selection can be specified even further to only include studies that explicitly focus on composite need satisfaction as typically defined by Kip Williams. Such a selection again provides support for the hypothesis that immediate fundamental need satisfaction is less moderated, Δ*d* = -0.18, 95% CI [-0.47, -0.11] (*k* = 15), than delayed need satisfaction, Δ*d* = -0.93, 95% CI [-1.67, -0.19] (*k* = 3). Note, however, that such a selection is based on 3 studies for delayed measures.

#### Composition

To inspect for structural and sampling effects of the studies, we ran mixed-effect models on the 120 ostracism effects, on both the first and the last measure. Due to listwise deletion, only 45 of 120 effect sizes remained on the first measure and 41 of 95 effect sizes for the last measure. The predictors in the mixed effects model were (1) country (US, other Western country, Asian, other), (2) proportion of males in the study, (3) mean age of the sample, (4) number of players in the game, (5) length of the game (≤ 5min, 5–10 min or > 10 min), (6) the number of throws in the game and (7) type of needs scale referenced (by assigning unique values for every unique reference).

On the first measure, this model (*k* = 45) showed clear residual heterogeneity after controlling for these structural- and sampling aspects of the studies, *Q*_*E*_ (33) = 449.52, *p* < .001, estimated τ_res_^2^ = 0.90, 95% CI [0.54, 1.59], but no overall moderation, *Q*_*M*_ (11) = 10.75, *p* = .465. The different types of need scales [11,20,21] did not significantly moderate effect sizes, showing psychometric convergence among the three scales. Inspecting the predictors individually also showed no indication for moderation (*p*s > .137; see Table 4).

**Table 4:** Meta regression coefficients for composition effects (first measure; k = 45).

	Estimate	(*SE*)	*Z*-value	*p*-value	95% CI Lowerbound	95% CI Upperbound
Intercept	-2.14	3.27	-1.89	0.058	-4.35	0.07
*Structural*						
Nr. of players	-0.22	1.05	-0.21	0.837	-2.28	1.85
Nr. of throws	0.03	0.02	1.49	0.137	-0.01	0.07
Ostracism <5 min	-	-	-	-	-	-
Ostracism 5–10 min	0.75	0.81	0.92	0.358	-0.84	2.34
Need scale = Williams (2000)	-	-	-	-	-	-
Need scale = Zadro et al. (2004)	-0.36	0.41	-0.88	0.381	-1.16	0.45
Need scale = Van Beest & Williams (2006)	0.07	0.54	0.13	0.894	-0.98	1.12
Need scale = Williams Zadro	-0.03	0.62	-0.04	0.965	-1.25	1.19
Need scale = Gonsalkorale & Williams (2007)	0.68	0.82	0.82	0.414	-0.94	2.30
*Sampling*						
Country = US	-	-	-	-	-	-
Country = Western	-0.42	0.36	-1.15	0.249	-1.13	0.29
Country = Asian	-0.30	1.13	-0.26	0.793	-2.51	1.92
Proportion male	1.54	1.09	1.42	0.156	-0.59	3.68
Mean age	-0.05	0.05	-0.97	0.332	-0.16	0.05

This can be interpreted as a standard regression formula. Empty rows represent reference categories.

On the last measure (*k* = 41; Table 5), no overall moderation was found, *Q*_*M*_ (11) = 6.00, *p* = .873, but heterogeneity did occur, *Q*_*E*_ (29) = 214.69, *p* < .0001. The number of players in the game significantly predicted the effects, *b* = 1.55, *p* = .047, 95% CI [0.2; 3.07], which would be interpreted as four players eliciting smaller ostracism effects, when compared to three players. The significance of this individual predictor should be interpreted carefully, as the omnibus moderation test showed no systematic decrease in heterogeneity. Overall, we found no strong evidence for moderation due to study or sample composition. We also conducted individual meta-regressions for each of the structural- and sampling variables. These individual analyses yield similar results as the overall analyses.

**Table 5:** Meta-regression coefficients for composition effects (last measure; k = 41).

	Estimate	(*SE*)	*Z*-value	*p*-value	95% CI Lowerbound	95% CI Upperbound
Intercept	-1.12	0.92	-1.21	0.227	-2.95	-0.70
*Structural*						
Nr. of players	1.55	0.78	1.98	0.047	0.02	3.07
Nr. of throws	0.01	0.02	0.59	0.556	-0.02	0.04
Ostracism <5 min	-	-	-	-	-	-
Ostracism 5–10 min	0.38	0.62	0.61	0.539	-0.83	1.59
Need scale = Williams (2000)	-	-	-	-	-	-
Need scale = Zadro et al. (2004)	-0.14	0.32	-0.44	0.658	-0.77	0.49
Need scale = Van Beest & Williams (2006)	-0.21	0.41	-0.51	0.613	-1.02	0.60
Need scale = Williams Zadro	-0.12	0.53	-0.22	0.826	-1.16	0.92
Need scale = Gonsalkorale & Williams (2007)	-0.07	0.65	-0.10	0.916	-1.33	1.20
*Sampling*						
Country = US	-	-	-	-	-	-
Country = Western	0.26	0.30	0.87	0.387	-0.33	0.86
Country = Asian	0.85	0.84	1.01	0.313	-0.80	2.49
Proportion male	0.29	0.83	0.35	0.730	-1.34	1.91
Mean age	-0.01	0.04	-0.25	0.806	-0.10	0.08

This can be interpreted as a standard regression formula. Empty rows represent reference categories.

#### Homogeneity

The analysis of the simple ostracism effect on the first measure showed that differences of underlying effects made up 93% of the variability in study outcomes. We performed an additional secondary analysis in a more homogenous subset of studies to better understand this heterogeneity. This subset only included typical Cyberball studies that involved three players in the game, 30 throws, and lasted less than five minutes. In addition, the homogeneous subset of typical Cyberball studies only involved measures of immediate fundamental needs (single or composite). Performing a meta-analysis on this homogeneous subset of 19 studies showed an *I* ^*2*^ value of 83%, indicating that 83% of the total variability can be attributed to heterogeneity in the effect sizes. We noted that the mean simple ostracism effect in these 19 studies was relatively strong and estimated at *d* = -2.05, 95% CI [-2.44, -1.65]. In other words, given that the heterogeneity remains large even in a homogeneous subset, suggests that the heterogeneity found in the overall analyses does not appear to be an artifact from the inclusion of different measures and the use of alternative Cyberball setups.

## Discussion

In this meta-analysis of Cyberball studies we estimated the average ostracism effect of the first and last dependent variable used in 120 Cyberball experiments. The primary hypotheses were (a) that the ostracism effect size would decrease from first to last measure and (b) that first measures would be less affected by cross-cutting variables than last measures. The secondary hypotheses tested whether the above generalizes across structural variables of the game, sample characteristics, or type of dependent variable used.

The results confirmed the hypothesis that the ostracism effect decreased from the first (*d* = -1.36) to the last measure (*d* = -.76), although this decline was not predicted by our estimation of duration between first and last measure. The results did not fully confirm the hypothesis that last measures are more strongly moderated than first measures. That is, our analysis of the experiments that included an experimentally controlled cross-cutting variable revealed that cross-cutting variables moderated both the first and last measure. In fact, visual inspection of the average estimated interaction effect sizes actually decreased in size from first (Δ*d* = -.46) to last (Δ*d* = -.19), although confidence intervals of these estimates did overlap.

To interpret the interactions it is important to recall (see Fig 3) that the *overall* ostracism effects are relatively large and operated similarly at both levels of the cross-cutting moderator variable. Moreover, when we compared the mean effects of the moderator variable *within* the two possible levels of ostracism factor (i.e., ostracized or include), results indicate a relatively weak *positive* effect within the ostracism level and a relatively weak *negative* effect within the inclusion level. To further explain the implication of the findings it may be fruitful to consider an example in which participants are ostracized or included by either an outgroup or an ingroup. In such a setting, our findings would thus suggest that the relative effect of ostracism compared to inclusion (i.e., the ostracism effect), is similar for both outgroup *and* ingroup conditions. Moreover, if one compares the effect of group status (outgroup vs. ingroup), one would predict that those ostracized by outgroup members would slightly benefit whereas those included by ingroup members would slightly be harmed. Taken together, these contrasts support the robustness of the ostracism effect. It is important to note that the simple effects in Fig 3 are averaged over studies, thus potentially subject to Simpson's paradox.

### Structural Aspects of Cyberball and Different Dependent Variables

The secondary analyses confirmed that the overall findings generalize to a large extent across structural aspects, sampling aspects and type of dependent variable.

#### Does gender of participants matter?

Previous research provided evidence for a difference in the ostracism effect across genders [17]. Our results indicated that, contrary to this, proportions of males and females did not significantly predict the mean effect size. In our coded studies, the mean proportion of males was approximately 39% (observed range: 0–100%).

#### Does age of participants matter?

Whereas previous research has indicated increased sensitivity to ostracism in younger age groups [18], we failed to find moderation of ostracism effects by mean age of the study samples. Coded studies had a mean sample age ranging from 10 through 32.5 years, with an average of approximately 20.5 years. This indicates that most of the research with Cyberball has been done on young adults, with relatively few or no studies investigating children, middle-aged participants, or senior citizens. More research could focus on specific (individual-level) age moderation of ostracism.

#### Does culture or country matter?

We found no indication that culture predicted the average effect size. In our coded studies, approximately 52% were from the United States, 45% from other Western countries (e.g., Australia, the Netherlands, Germany), and 3% from Asian countries. Our analyses used the United States as reference category. We note that the low prevalence of Asian countries might cause a lack of power and that we cannot definitively state there is no difference between Western and Asian responses to ostracism. We can state that there is no systematic difference in the ostracism response for Western countries and the United States.

#### Does number of players matter?

In the studies included in this meta-analysis, approximately 89% of the studies used the three-player version of Cyberball and 11% used the four-player version of Cyberball. Average ostracism effects differed between these subsets, with smaller predicted effects in the four-player setting, but we are hesitant to interpret this due to a nonsignificant omnibus test for the predictive model (see ‘Composition‘ in the results section). Preferably, this moderator of the ostracism effect in Cyberball should be subject to further work in which the number of players is experimentally varied.

#### Does number of throws or length of the study matter?

We considered the length of Cyberball in two ways. We coded the number of ball tosses and estimated the length of the study. Of the coded studies, 60% used 30 throws, 11% used 40 throws, 8% used 20 throws, 4% used 60 throws, and 2% for both 15 and 24 throws. Other categories ranging from 10 through 200 make up the remaining percentages, each making up 1%. Only 2 out of 120 studies were estimated to last longer than 5 minutes. Our results indicated the mean ostracism effect was *not* reliably predicted to be different across different lengths of the study or the different number of total throws in the omnibus test. The single meta-regression on ball tosses suggested it may predict the effect size of the first measure. As above, we are hesitant to interpret this, but do note that increasing ball tosses may be more associated with a diffused ostracism effect than with an increased ostracism effect.

#### Does type of dependent variable matter?

Secondary analyses also showed that the majority of the results were robust across subsets of dependent measures and the overall set of dependent measures (see Fig 3). Exceptions were interpersonal measures showing relatively weaker ostracism effects on the first measure when compared to the other subsets. This suggests that psychological effects of ostracism are large, but that this effect might be smaller for interpersonal behaviors. On top of this, interpersonal measures also show more moderation, suggesting that interpersonal behaviors caused by ostracism are more easily moderated by cross-cutting factors. Additionally, we estimated interactions for the measure subsets interpersonal (i.e., measures relating to others), intrapersonal (measures relating to the self), fundamental needs, model (i.e., first measure is reflexive and last measure is reflective), and an overlap of the latter two subsets. For all but two, these subsets showed that measures taken at the first time point were moderated more strongly than the measures taken last. Finally, the analyses including only fundamental needs showed that moderation was larger at the last time point, when compared to the first time point. This result is crucial, as Williams [11] specifically predicted this pattern for fundamental needs.

### Williams’s Model of Ostracism: Supported or Not?

Regarding the test of Williams’s [11] model, there are several important observations and limitations. First, Williams proposed fundamental need threat as a result of even a brief episode of ostracism. This was supported by the meta-analysis. Moreover, moderation is predicted to occur in the reflective stage, when the context and meaning of the ostracism event can be appraised. This was also supported in the present meta-analysis. The final stage of Williams’s model—resignation—is outside the aims of the present meta-analysis, because it requires long-term exposure to ostracism.

The proposition that appears to lack support from this meta-analysis is that reflexive reactions to ostracism are more resistant to moderation than reflective reactions. Across the board, our results indicate there is more moderation of ostracism effects on the first time point than on the last time point. However, there are two limitations to this conclusion. Firstly, Williams specifically refers to physiological, online, or immediate retrospective reports to assess reflexive reactions. In many instances in this meta-analysis, the first reaction is not isomorphic with reflexive measures. Anything taken after the game, or assessed by wording indicating present state (rather than the participants’ state during the game), is not assumed to be reflexive, nor predicted to be resistant to moderation. Secondly, Williams’s proposition is restricted to fundamental needs only. Indeed, our specific analyses involving only studies that employed measures of immediate and delayed fundamental need satisfaction corroborated the model prediction that there is more moderation on the last time point, than on the first time point.

Because of this quantitative difference in moderation across measures, we encourage direct testing of this time difference in moderation as predicted by Williams [11], just as the study by Bernstein and Claypool [39] was a direct, experimental test of a finding by Gerber and Wheeler [13]. However, the mean size of the interaction effect in out meta-analysis was quite small, raising power issues for future studies. Using our estimated interaction effects to determine sample size under a power of .8, a sample size of 2186 would be necessary to have sufficient power on both time points. We used G*Power 3.1.7 to calculate this between-subjects interaction effect (*F*-test, fixed effects, .8 power); with *k* = 4 and the smaller interaction (last time point; numerator *df* = *k—*1). The effect size Δ*d* was transformed in to *f* by means of √[*d* ^*2*^/(2*k*)], resulting in *f* = .0707. Note that the mean sample size in full factorial designs in our meta-analysis is 110, showing that the mean power in these studies is .08 to detect an *interaction* at the last time point (notably, power for the standard ostracism effect is highly sufficient in the included studies, due to the large effect). A large Mechanical Turk study is feasible and could provide the sample needed. Additional ways of increasing power are by reducing error on the measurements by using validated psychometric scales.

#### Changes to the need-threat model of ostracism

As a result of our findings, we suggest that the temporal need-threat model of ostracism should be modified. Firstly, it should be recognized that there is potential for moderation in the reflexive stage, where immediate measures of impact tap into participants’ reactions during the game. If factors can reduce physical pain and distress, like for instance acetaminophen [40] ([40] was not included in the meta-analysis, because we were not able to retrieve all information) or transcranial magnetic stimulation [41], or if certain populations are less likely to feel pain (e.g., those higher in schizotypal personality disorder [42,43]), then we would also expect moderation of immediate measures of distress. Secondly, our results may suggest important issues related to the timing of measuring ostracism effects by way of the ordinal differences. Specifically, time passed after the ostracism episode occurred is likely to affect the extent immediate distress measures will be subject to moderation. For example, if researchers wait long enough before administering the immediate need satisfaction measures (e.g., “playing the game made me feel insecure”), it becomes more likely that all participants will have recovered from the negative impact of ostracism, thus resulting in a homogeneous (and highly satisfied) between-group result. Thus, differences in recovery from ostracism based upon social-situational factors and/or personality differences, if any, occur somewhere between initial pain and final recovery. It is difficult to predict exactly when that time period is. Zadro et al. [44] report delayed recovery by those high in social anxiety 45-minutes later. Other studies show full recovery within 5–10 minutes. Future research needs to examine the time course more carefully, to determine if and when moderation occurs in delayed measures.

### Limitations

Within the current meta-analysis there are several limitations. One potential limitation is that our testing of differences between first and last measure was indirect. We compared confidence intervals to evaluate whether the effects were different. A direct test would provide more conclusive evidence on whether or not the effects are indeed equal or different across the first and last measurements. Note, however, that a direct test requires correlations between the measurements for every study, every condition, and every type of different variable. This information was not given in the vast majority of the papers and we anticipated that a direct request for such information would suffer from the problem of low response rates [45] which would in turn lower the sample size of the meta-analysis and thus the ability to effectively test our hypotheses.

A second potential limitation is that the random (non-systematic) heterogeneity in the effect sizes poses a problem for the power of finding moderator effects [24]. This could pose the problem that several of the non-effects found are actually there, but not detected (Type II errors). However, our subset analysis of typical Cyberball studies—3 players games involving 30 ball tosses, lasting less than five minutes, with immediate fundamental need satisfaction as dependent variable—still showed substantial variability in the effect sizes: *I* ^*2*^ = 83%. This indicates that the effects are quite variable to begin with and makes it unlikely that the overall effects are misrepresented.

Also, we did not observe that our estimation of time predicted the ostracism effect on the last measure. This null-effect may be a reality but could also be caused by the fact that the (random) heterogeneity in the effect sizes may have been too large to find moderation by time. This cannot be counteracted in the current dataset and remains a limitation. Second, imprecise reporting of the measures in the papers may have led to inaccurate time estimations. To counteract this imprecise reporting of measures, authors could be contacted, but this also poses new problems (i.e., nonresponse, or authors might not be willing to admit that measures were left out in the paper [46]).

Importantly, we did observe that the confidence intervals of both the first and last measure did not overlap, suggesting that there is a difference in effect size between first and last measure. The question then is whether this difference is indeed caused by time of measurement or in part caused by the type of measurement used across the two different time points. This explanation can be addressed by inspecting whether the composition of measures is different across time points. On the first measure 0.84 was intrapersonal self-report, 0.02 was intrapersonal physiological, 0.01 was intrapersonal other, 0.08 was interpersonal anti-social, 0.03 was interpersonal pro-social, and 0.01 interpersonal other. On the last measure 0.79 was intrapersonal self-report, 0.04 was intrapersonal physiological, 0.02 was intrapersonal other, 0.05 was interpersonal anti-social, 0.08 was interpersonal pro-social, and 0.01 was interpersonal other. This shows that the different types of dependent variables are similarly distributed across time points (maximum discrepancy of 4.9 percentage points). Substantive differences in proportions of measures across time points are minimal and thus form an unlikely driving force for our findings.

A third limitation is that this paper only summarized the results of the measures included in the studies. However obvious this might be, it should be pointed out, because the validity of the conclusions are reliant on the validity of the measures. Most prominently represented in the current meta-analysis are the fundamental need measures, which have no proper psychometric validation up-to-date, notwithstanding their wide use. Other kinds of included measures possibly also lack proper validation and one has been openly criticized (e.g., the Hot Sauce aggression paradigm [47]).

## Conclusion

Our meta-analysis of 120 Cyberball studies extends the temporal need-threat model of ostracism. We observed that the average effect size approaches 1.5 standard deviations and that this average effect size is not affected by the composition of the sample used (i.e., age, gender, country of origin) nor by structural aspects of the game (i.e., number of ball tosses, duration, players). We also observed that findings are relatively robust across the typical dependent variables that are used in Cyberball and that the overall effect size decreases from first to last measure. Importantly, we also observed that first measures can be moderated by cross-cutting variables and that only fundamental needs measures show stronger moderation for the last measures as opposed to the first measure taken in the studies. The moderation analyses by cross-cutting variables also revealed that the interaction effects sizes are considerably smaller than the direct inclusion vs. ostracism effect size. This revealed that the typical Cyberball study has enough power to detect main effects, but should substantially increase sample size to study theoretically relevant interactions. Intriguingly, we also observed that effect sizes were rather heterogeneous even when we limited our analysis to a very homogenous subset of studies. This indicates that there are potentially relevant moderators that have yet not been discovered. We invite fellow researchers to reanalyze our data (osf.io/ht25n) and test new hypotheses, and to further expand our knowledge of ostracism with Cyberball.

## Supporting Information

S1 File Data package. Contains data and the R analysis script.
(ZIP)

S2 File Full reference list meta-analysis studies. Contains the full reference list of the studies included in the meta-analysis.
(DOCX)

S3 File Scatterplot of the effects in hypotheses 1 and 2 and estimated time. (TIFF)

S4 File Fig 3 subset lists. Contains the lists of what studies that were in the meta-analysis are included in computing the effects for the different panels.
(XLSX)

S5 File PRISMA checklist. (DOC)

S6 File Effect size formulae. (DOCX)
