# Supplementary material for: The Ordinal Effects of Ostracism: A Meta-Analysis of 120 Cyberball Studies
Source: PLoS One. 2015 May 29;10(5):e0127002. doi: 10.1371/journal.pone.0127002 (PMC4449005; doi:10.1371/journal.pone.0127002)
Supplement: S2 File — Contains the full reference list of the studies included in the meta-analysis. (DOCX) [file pone.0127002.s002.docx]

Reference list of included studies

Alvares, G. A., Hickie, I. B., & Guastella, A. J. (2010). Acute effects of intranasal oxytocin on subjective and behavioral responses to social rejection. *Experimental and Clinical Psychopharmacology*, *18*, 316–321. doi: 10.1037/a0019719

Ambrosini, E., Blomberg, O., Mandrigin, A., & Costantini, M. (2014). Social exclusion modulates pre-reflective interpersonal body representation. *Psychological Research, 78,* 28-36. doi: 10.1007/s00426-012-0476-2

Aydin, N., Krueger, J. I., Fischer, J., Hahn, D., Kastenmüller, A., Frey, D., & Fischer, P. (2012). “Man’s best friend:” How the presence of a dog reduces mental distress after social exclusion. *Journal of Experimental Social Psychology*, *48*, 446–449. doi: 10.1016/j.jesp.2011.09.011

Banki, S. (2012). *How much or how many? Partial ostracism and its consequence* (Doctoral dissertation). Retrieved from T-Space at The University of Toronto Libraries (http://hdl.handle.net/1807/32659 ).

Bastian, B., & Haslam, N. (2010). Excluded from humanity: The dehumanizing effects of social ostracism. *Journal of Experimental Social Psychology*, *46*, 107–113. doi: 10.1016/j.jesp.2009.06.022

Bernstein, M. J., & Claypool, H. M. (2012a). Social exclusion and pain sensitivity: Why exclusion sometimes hurts and sometimes numbs. *Personality & Social Psychology Bulletin*, *38*, 185–196. doi: 10.1177/0146167211422449

Bernstein, M. J., & Claypool, H. M. (2012b). Not all social exclusions are created equal: Emotional distress following social exclusion is moderated by exclusion paradigm. *Social Influence*, *7*, 113–130. doi: 10.1080/15534510.2012.664326

Bernstein, M. J., Sacco, D. F., Young, S. G., Hugenberg, K., & Cook, E. (2010a). Being “in” with the in-crowd: The effects of social exclusion and inclusion are enhanced by the perceived essentialism of ingroups and outgroups. *Personality & Social Psychology Bulletin*, *36*, 999–1009. doi: 10.1177/0146167210376059

Bernstein, M. J., Sacco, D. F., Young, S. G., Hugenberg, K., & Cook, E. (2010b). *Unpublished study of “Being ‘in’ with the crowd” paper*. Unpublished study, Pennstate Abington, PA.

Boyes, M. E., & French, D. J. (2009). Having a Cyberball: Using a ball-throwing game as an experimental social stressor to examine the relationship between neuroticism and coping. *Personality and Individual Differences*, *47*, 396–401. doi: 10.1016/j.paid.2009.04.005

Brochu, P. M., Garcia, D. M., Smith, H. J., & Esses, V. M. (2013). *Ironic Effects of Ambiguous Weight Stigmatization on the Regulation of Eating Behavior*. Manuscript in preparation, Nova Southeastern University, Florida, FL.

Brown, C. M., Young, S. G., Sacco, D. F., Bernstein, M. J., & Claypool, H. M. (2009). Social Inclusion Facilitates Interest in Mating. *Evolutionary Psychology*, *7*, 11–27.

Carter, B. E. (2008). *When does ostracism decrease self-regulation?* (master’s thesis). Retrieved from Scholarworks (http://scholarworks.montana.edu/xmlui/handle/1/1047).

Carter-Sowell, A. R., Wesselmann, E. C., Wirth, J. H., Law, A. T., Chen, Z., Kosasih, M. W., & Van der Lee, R. (2010). Strides for belonging trump strides for superiority: Effects of being ostracized for being superior or inferior to the others. *The Journal of Individual Psychology*, *66*, 68–92.

Carter-Sowell, A. R, Chen, Z., & Williams, K. D. (2008). Ostracism increases social susceptibility. *Social Influence*, *3*, 143–153. doi: 10.1080/15534510802204868

Chen, Z., DeWall, C. N., Poon, K.-T., & Chen, E.-W. (2012). When destiny hurts: Implicit theories of relationships moderate aggressive responses to ostracism. *Journal of Experimental Social Psychology*, *48*, 1029–1036. doi: 10.1016/j.jesp.2012.04.002

Chernyak, N., & Zayas, V. (2010). Being excluded by one means being excluded by all: Perceiving exclusion from inclusive others during one-person social exclusion. *Journal of Experimental Social Psychology*, *46*, 582–585. doi: 10.1016/j.jesp.2010.01.004

Chow, R. M., Tiedens, L. Z., & Govan, C. L. (2008). Excluded emotions: The role of anger in antisocial responses to ostracism. *Journal of Experimental Social Psychology*, *44*, 896–903. doi: 10.1016/j.jesp.2007.09.004

Chrisp, J. A. L. (2012). *Exploring the role of belonging in intergroup discrimination* (master’s thesis)*.* Retrieved from Otago University Research Archive (http://hdl.handle.net/10523/2662).

Coyne, S. M., Gundersen, N., Nelson, D. A., & Stockdale, L. (2011). Adolescents’ prosocial responses to ostracism: An experimental study. *The Journal of Social Psychology*, *151*, 657–661. doi: 10.1080/00224545.2010.522625

De Waal-Andrews, W., & Van Beest, I. (2012). When you don’t quite get what you want: Psychological and interpersonal consequences of claiming inclusion. *Personality and Social Psychology Bulletin*, *38*, 1367–1377. doi: 10.1177/0146167212450463

DeBono, A. (2013). *Understanding Maladaptive Responses to Social Exclusion: Aggression with an Audience*. Manuscript in preparation, Winston-Salem State University, North Carolina.

DeBono, A., & Muraven, M. (2013). *Personality traits that reduce and exacerbate aggression from social exclusion when depleted*. Manuscript in preparation, Winston-Salem State University, North Carolina.

Dietrich, D., Hawkinson, K., & Palo, A. (2010, January). Self-monitoring as a moderating factor of aggressive responses to ostracism. Poster presented at Society for Personality and Social Psychology conference, Las Vegas, NV.

Duclos, R., Wan, E. W., & Jiang, Y. (2013). Show me the honey! Effects of social exclusion on financial risk-taking. *Journal of Consumer Research*, *40*, 122–135. doi: 10.1086/668900

Eisenberger, N. I., Jarcho, J. M., Lieberman, M. D., & Naliboff, B. D. (2006). An experimental study of shared sensitivity to physical pain and social rejection. *Pain*, *126*, 132–138. doi: 10.1016/j.pain.2006.06.024

Fayant, M. P., Lantian, A., Muller, D., & Hartgerink, C.H.J. (in press). Is ostracism by a despised group suffering? A replication of Gonsalkorale and Williams (2007). *Social Psychology*.

Floor, L. (2007). *De effecten van groepslidmaatschap op ostracisme en pesten. Wat is erger: genegeerd of gepest worden? [The effects of group membership on ostracism and bullying. What is worse: being ignored or being bullied?]*. University of Leiden, the Netherlands.

Gallardo-Pujol, D., Andrés-Pueyo, A., & Maydeu-Olivares, A. (2012). MAOA genotype, social exclusion and aggression: An experimental test of a gene-environment interaction. *Genes, Brain, and Behavior*. doi: 10.1111/j.1601-183X.2012.00868.x

Gan, Y., & Liu, J. (2012). The mechanism by which interpersonal coping flexibility influences self-esteem. *The Psychological Record*, *62*, 735–746.

Garczynski, A. M., Brown, C. M., & Harvey, R. D. (2013, January). Temporal perspective moderates self-reported reactions to social rejection. Poster presented at Society for Personality and Social Psychology conference, New Orleans, LA.

Geniole, S. N., Carré, J. M., & McCormick, C. M. (2011). State, not trait, neuroendocrine function predicts costly reactive aggression in men after social exclusion and inclusion. *Biological Psychology*, *87*, 137–145. doi: 10.1016/j.biopsycho.2011.02.020

Gerber, J. P., Williams, K. D., & Wheeler, L. (2013). *Clarifying the relationship between ostracism and relational devaluation.* Manuscript in preparation, Gordon College, Wenham.

Gonsalkorale, K., & Williams, K. D. (2007). The KKK won’t let me play: Ostracism even by a despised outgroup hurts. *European Journal of Social Psychology*, *37*, 1176–1186. doi: 10.1002/ejsp.392

Goodwin, S. A., Williams, K. D., & Carter-Sowell, A. R. (2010). The psychological sting of stigma: The costs of attributing ostracism to racism. *Journal of Experimental Social Psychology*, *46*, 612–618. doi: 10.1016/j.jesp.2010.02.002

Greitemeyer, T., Fischer, P., & Kastenmüller, A. (2012). The effects of social exclusion on confirmatory information processing. *European Journal of Social Psychology*, *42*, 462–469. doi: 10.1002/ejsp.1851

Gruijters, S. (2013). [Social connection and anthropomorphism]. Unpublished raw data.

Hackenbracht, J., & Gasper, K. (2013). I’m All Ears: The Need to Belong Motivates Listening to Emotional Disclosure. *Journal of Experimental Psychology, 49,* 915-921. doi: 10.1016/j.jesp.2013.03.014

Hawes, D. J., Zadro, L., Fink, E., Richardson, R., O’Moore, K., Griffiths, B., Dadds, M. R., et al. (2012). The effects of peer ostracism on children’s cognitive processes. *European Journal of Developmental Psychology*, *9*, 599–613. doi: 10.1080/17405629.2011.638815

Hellmann, J. H., & Echterhoff, G. (2013). *Ostracism and Facebook*. Manuscript in preparation, University of Münster, Germany.

Hess, Y. D., & Pickett, C. L. (2010). Social rejection and self- versus other-awareness. *Journal of Experimental Social Psychology*, *46*, 453–456. doi: 10.1016/j.jesp.2009.12.004

Hess, Y., & Pickett, C. (2011, January). I’ll stick with my idea: exclusion increases socially dominant behaviors. Poster presented at Society for Personality and Social Psychology conference, San Antonio, TX.

Horn, N. R. (2010). *Social exclusion and the bitter coldness of rejection: can drinking a warm drink after being socially excluded counterbalance the negative effects?* (bachelor’s thesis). Retrieved from DSpace Repository (https://dspace.washcoll.edu/handle/10090/19133?show=full).

Ijzerman, H., Gallucci, M., Pouw, W. T. J. L., Weiβgerber, S. C., Van Doesum, N. J., & Williams, K. D. (2012). Cold-blooded loneliness: social exclusion leads to lower skin temperatures. *Acta Psychologica*, *140*, 283–288. doi: 10.1016/j.actpsy.2012.05.002

Jamieson, J. P., Harkins, S. G., & Williams, K. D. (2010). Need threat can motivate performance after ostracism. *Personality & Social Psychology Bulletin*, *36*, 690–702. doi: 10.1177/0146167209358882

Johnson, C. (2010). *Personality and ostracism: do hope, optimism, and forgiveness moderate the effects of social exclusion?* (master’s thesis). Retrieved from Purdue University e-Pubs (AAI1489002).

Kassner, M. P., Dongning, R., Law, A. T., & Williams, K. D. (2013). *Effects of Mental Visualization and Degraded Presentation on Detection and Influence of Ostracism*. Manuscript in preparation, Purdue University, Purdue, IN.

Kassner, M. P., Wesselmann, E. D., Law, A. T., & Williams, K. D. (2012). Virtually ostracized: Studying ostracism in immersive virtual environments. *Cyberpsychology, Behavior and Social Networking*, *15*, 399–403. doi: 10.1089/cyber.2012.0113

Kerr, N. L., Seok, D.-H., Poulsen, J. R., Harris, D. W., & Messé, L. A. (2008). Social ostracism and group motivation gain. *European Journal of Social Psychology*, *38*, 736–746. doi: 10.1002/ejsp.499

Kesting, M.-L., Bredenpohl, M., Klenke, J., Westermann, S., & Lincoln, T. M. (2012). The impact of social stress on self-esteem and paranoid ideation. *Journal of Behavior Therapy and Experimental Psychiatry*, *44*, 122–128. doi: 10.1016/j.jbtep.2012.07.010

Knowles, M. (2010, January). Use of social media in the service of belonging needs. Poster presented at Society for Personality and Social Psychology conference, Las Vegas, NV.

Knowles, M., & Caroll, C. (2012, January). Shifting perspectives: the impact of social rejection and acceptance on perspective-taking. Poster presented at Society for Personality and Social Psychology conference, San Diego, CA.

Krijnen, J. (*n.d.*). Research Report “BP29 Complete.” Tilburg University, the Netherlands.

Krill, A. L., Platek, S. M., & Wathne, K. (2008). Feelings of control during social exclusion are partly accounted for by empathizing personality. *Personality and Individual Differences*, *45*, 684–688. doi: 10.1016/j.paid.2008.07.016

Lakin, J. L., Chartrand, T. L., & Arkin, R. M. (2008). I am too just like you: Nonconscious mimicry as an automatic behavioral response to social exclusion. *Psychological science*, *19*, 816–822. doi: 10.1111/j.1467-9280.2008.02162.x

Lau, G., Moulds, M. L., & Richardson, R. (2009). Ostracism: How much it hurts depends on how you remember it. *Emotion*, *9*, 430–434. doi: 10.1037/a0015350

Lustenberger, D. E., & Jagacinski, C. M. (2010). Exploring the Effects of Ostracism on Performance and Intrinsic Motivation. *Human Performance*, *23*, 283–304. doi: 10.1080/08959285.2010.501046

MacDonald, G. (2008). Use of pain threshold reports to satisfy social needs. *Pain Research & Management*, *13*, 309–319.

McDonald, M. M., & Donnellan, B. M. (2012). Is ostracism a strong situation? The influence of personality in reactions to rejection. *Journal of Research in Personality*, *46*, 614–618. doi: 10.1016/j.jrp.2012.05.008

Nordgren, L. F., Banas, K., &MacDonald, G. (2011). Empathy gaps for social pain: Why people underestimate the pain of social suffering. *Journal of Personality and Social Psychology*, *100*, 120–128. doi: 10.1037/a0020938

Nordgren, L. F., McDonnell, M.-H.M., & Loewenstein, G. (2011). What constitutes torture?: Psychological impediments to an objective evaluation of enhanced interrogation tactics. *Psychological Science*, *22*, 689–694. doi: 10.1177/0956797611405679

O’Brien, E., Ellsworth, P. C., & Schwarz, N. (2012). Today’s misery and yesterday's happiness: Differential effects of current life-events on perceptions of past wellbeing. *Journal of Experimental Social Psychology*, *48*, 968–972. doi: 10.1016/j.jesp.2012.02.018

Oberleitner, D. E. (2012). *Accessibility for aggression and negative self-views following ostracism*. Retrieved from Wayne State University Dissertations (Paper 554).

Peterson, C. K., Gravens, L. C., & Harmon-Jones, E. (2011). Asymmetric frontal cortical activity and negative affective responses to ostracism. *Social Cognitive and Affective Neuroscience*, *6*, 277–285. doi: 10.1093/scan/nsq027

Pharo, H., Gross, J., Richardson, R., & Hayne, H. (2011). Age-related changes in the effect of ostracism. *Social Influence*, *6*, 22–38. doi: 10.1080/15534510.2010.525852

Plaisier, X. S., & Konijn, E. A. (2013). Rejected by Peers-Attracted to Antisocial Media Content: Rejection-Based Anger Impairs Moral Judgment Among Adolescents. *Developmental Psychology, 49,* 1165-1173. doi: 10.1037/a0029399

Ramirez, M. C. (2009). *The influence of contingent self-esteem and self-esteem variability on reactions to ostracism* (master’s thesis). Retrieved from DSpace University of Texas at Arlington. (http://dspace.uta.edu/bitstream/handle/10106/2034/Ramirez_uta_2502M_10456.pdf?sequence=1).

Ren, D., & Williams, K. D. (2012, January). Self-construals matter when coping with ostracism: but not when experiencing it. Poster presented at Society for Personality and Social Psychology conference, San Diego, CA.

Renneberg, B., Herm, K., Hahn, A., Staebler, K., Lammers, C.-H., & Roepke, S. (2012). Perception of social participation in borderline personality disorder. *Clinical Psychology & Psychotherapy, 19,* 473-480. doi: 10.1002/cpp.772

Riva, P., Wirth, J. H., & Williams, K. D. (2011). The consequences of pain: The social and physical pain overlap on psychological responses. *European Journal of Social Psychology*, *41*, 681–687. doi: 10.1002/ejsp.837

Ruggieri, S., Bendixen, M., Gabriel, U., & Alsaker, F. (2013). *Do victimization experiences accentuate reactions to ostracism? An experiment using Cyberball*. Manuscript submitted for publication, Universität Bern, Switzerland.

Ruggieri, S., Bendixen, M., Gabriel, U., & Alsaker, F. (2013). Cyberball: The impact of ostracism on early adolescents’ well-being. *Swiss Journal of Psychology, 72,* 103-109. doi: 10.1024/1421-0185/a000103

Sacco, D. F., Wirth, J. H., Hugenberg, K., Chen, Z., & Williams, K. D. (2011). The world in black and white: Ostracism enhances the categorical perception of social information. *Journal of Experimental Social Psychology*, *47*, 836–842. doi: 10.1016/j.jesp.2011.03.001

Salvy, S.-J., Bowker, J. C., Nitecki, L. A., Kluczynski, M. A., Germeroth, L. J., & Roemmich, J. N. (2010). Impact of simulated ostracism on overweight and normal-weight youths’ motivation to eat and food intake. *Appetite*, *56*, 39–45. doi: 10.1016/j.appet.2010.11.140

Salvy, S.-J., Bowker, J. C., Nitecki, L. A., Kluczynski, M. A., Germeroth, L. J., & Roemmich, J. N. (2012). Effects of ostracism and social connection-related activities on adolescents’ motivation to eat and energy intake. *Journal of Pediatric Psychology*, *37*, 23–32. doi: 10.1093/jpepsy/jsr066

Schaafsma, J., & Williams, K. D. (2012).Exclusion, intergroup hostility, and religious fundamentalism. *Journal of Experimental Social Psychology*, *48*, 829–837. doi: 10.1016/j.jesp.2012.02.015

Segovia, K. Y., & Bailenson, J. N. (2012). Virtual imposters: Responses to avatars that do not look like their controllers. *Social Influence*, *7*, 285–303. doi: 10.1080/15534510.2012.670906

Staebler, K., Renneberg, B., Stopsack, M., Fiedler, P., Weiler, M., & Roepke, S. (2011). Facial emotional expression in reaction to social exclusion in borderline personality disorder. *Psychological Medicine*, *41*, 1929–1938. doi: 10.1017/S0033291711000080

Stillman, T. F., Baumeister, R. F., Lambert, N. M., Crescioni, A. W., Dewall, C. N., & Fincham, F. D. (2009). Alone and Without Purpose: Life Loses Meaning Following Social Exclusion. *Journal of Experimental Social Psychology*, *45*, 686–694. doi: 10.1016/j.jesp.2009.03.007

Stock, M. L., Gibbons, F. X., Walsh, L. A., & Gerrard, M. (2011). Racial identification, racial discrimination, and substance use vulnerability among African American young adults. *Personality and Social Psychology Bulletin*, *37*, 1349–1361. doi: 10.1177/0146167211410574

Van Beest, I., Williams, K. D., & Van Dijk, E. (2011). Cyberbomb: Effects of being ostracized from a death game. *Group Processes & Intergroup Relations*, *14*, 581–596. doi: 10.1177/1368430210389084

Van Beest, I., Carter-Sowell, A. R., Van Dijk, E., & Williams, K. D. (2012). Groups being ostracized by groups: Is the pain shared, is recovery quicker, and are groups more likely to be aggressive? *Group Dynamics: Theory, Research, and Practice*, *16*, 241–254. doi: 10.1037/a0030104

Van Beest, I., Meijs, M., Van Kleef, G., Beersma, B., & Homan, A. (2013). *Partial Ostracism: Partial Pain, Partial Revenge, Partial Recovery*. In Preparation.

Van Beest, I,, & Williams, K. D. (2006). When inclusion costs and ostracism pays, ostracism still hurts. *Journal of Personality and Social Psychology*, *91*, 918–928. doi: 10.1037/0022-3514.91.5.918

Van Dijk, W. W., & Williams, K. D. (2013). [Social exclusion and schadenfreude.] Unpublished raw data.

Webb, T. L., Harris, P., & McAtamney, K. (2013). *Self-affirmation moderates the negative effect of ostracism on perceptions of control, but not in predictable ways*. Unpublished manuscript, University of Sheffield, United Kingdom.

Weik, U., Maroof, P., Zöller, C., & Deinzer, R. (2010). Pre-experience of social exclusion suppresses cortisol response to psychosocial stress in women but not in men. *Hormones and Behavior*, *58*, 891–897. doi: 10.1016/j.yhbeh.2010.08.018

Wesselmann, E. D., Bagg, D., & Williams, K. D. (2009). “I feel your pain”: The effects of observing ostracism on the ostracism detection system. *Journal of Experimental Social Psychology*, *45*, 1308–1311. doi: 10.1016/j.jesp.2009.08.003

Wesselmann, E. D., Wirth, J. H., Mroczek, D. K., & Williams, K. D. (2012). Dial a feeling: Detecting moderation of affect decline during ostracism. *Personality and Individual Differences*, *53*, 580–586. doi: 10.1016/j.paid.2012.04.039

Williams, K D, Cheung, C. K., & Choi, W. (2000). Cyberostracism: Effects of being ignored over the Internet. *Journal of Personality and Social Psychology*, *79*, 748–762. doi: 10.1037/0022-3514.79.5.748

Williams, K. D, Govan, C. L., Croker, V., Tynan, D., Cruickshank, M., & Lam, A. (2002). Investigations into differences between social- and cyberostracism. *Group Dynamics: Theory, Research, and Practice*, *6*, 65–77. doi: 10.1037//1089-2699.6.1.65

Wirth, J. H., & Williams, K. D. (2009). ‘They don’t like our kind’: Consequences of being ostracized while possessing a group membership. *Group Processes & Intergroup Relations*, *12*, 111–127. doi: 10.1177/1368430208098780

Wirth, J. H., Lynam, D. R., & Williams, K. D. (2010). When social pain is not automatic: Personality disorder traits buffer ostracism’s immediate negative impact. *Journal of Research in Personality*, *44*, 397–401. doi: 10.1016/j.jrp.2010.03.001

Zadro, L., Boland, C., & Richardson, R. (2006). How long does it last? The persistence of the effects of ostracism in the socially anxious. *Journal of Experimental Social Psychology*, *42*, 692–697. doi: 10.1016/j.jesp.2005.10.007

Zadro, L., Williams, K. D., & Richardson, R. (2004). How low can you go? Ostracism by a computer is sufficient to lower self-reported levels of belonging, control, self-esteem, and meaningful existence. *Journal of Experimental Social Psychology*, *40*, 560–567. doi: 10.1016/j.jesp.2003.11.006

Zhong, C.-B., & Leonardelli, G. J. (2008). Cold and lonely: Does social exclusion literally feel cold? *Psychological Science*, *19*, 838–842. doi: 10.1111/j.1467-9280.2008.02165.x

Zöller, C., Maroof, P., Weik, U., & Deinzer, R. (2010). No effect of social exclusion on salivary cortisol secretion in women in a randomized controlled study. *Psychoneuroendocrinology*, *35*, 1294–1298. doi: 10.1016/j.psyneuen.2010.02.019

Zwolinski, J. (2012). Psychological and neuroendocrine reactivity to ostracism. *Aggressive Behavior*, *38*, 108–125. doi:10.1002/ab.21411
