# Supplementary figures and images for: The Ordinal Effects of Ostracism: A Meta-Analysis of 120 Cyberball Studies
Source: PLoS One. 2015 May 29;10(5):e0127002. doi: 10.1371/journal.pone.0127002 (PMC4449005; doi:10.1371/journal.pone.0127002)

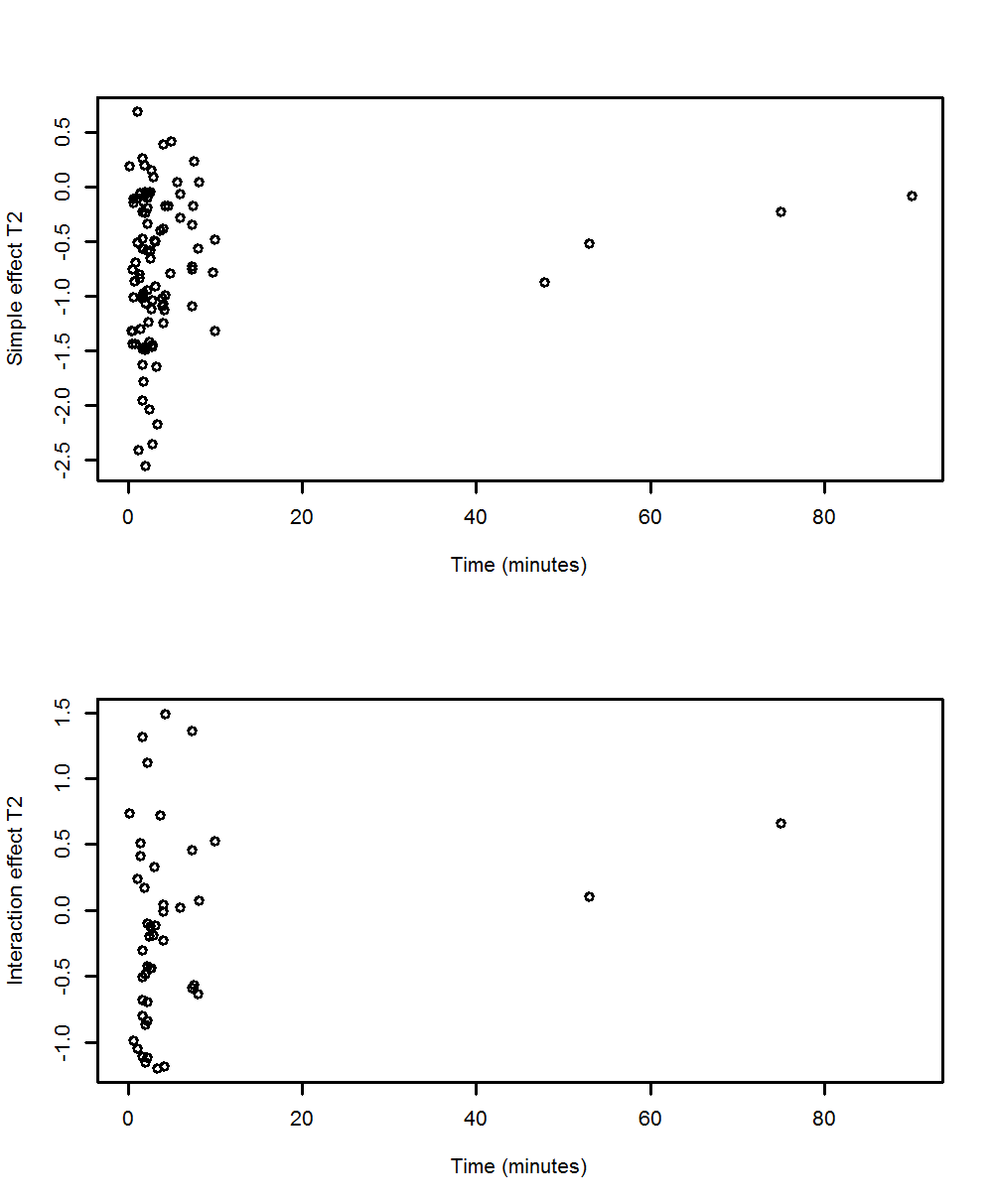

Supplement: S3 File — (TIFF) [file pone.0127002.s003.tiff]
