# Supplementary material for: The Ordinal Effects of Ostracism: A Meta-Analysis of 120 Cyberball Studies
Source: PLoS One. 2015 May 29;10(5):e0127002. doi: 10.1371/journal.pone.0127002 (PMC4449005; doi:10.1371/journal.pone.0127002)
Supplement: S6 File — (DOCX) [file pone.0127002.s006.docx]

All formulae reported below originate from the chapter by Michael Borenstein (2009). Hedges’ *g* was calculated as

$$g=d\left(1-\frac{3}{4df_{w}-9}\right)$$

where *d* is the standardized main effect and *df_w_* is the number of conditions minus 1. For the standardized interaction effect *d* was calculated as

where the first term in the numerator is the ostracism effect and the second term is the ostracism effect in the moderator conditions. When transformed to a squared correlation coefficient, this Δ*d* corresponds to the partial eta-squared of the interaction. Sampling variance of *g* was calculated by multiplying the sampling variance of *d* by the squared correction factor, that is

$$s_{g}^{2}=\left(1-\frac{3}{4df_{w}-9}\right)^{2}s_{d}^{2}$$

where the sampling variance of the interaction was calculated as the sum of the sampling variances of both the simple main effects.

**Reference**

Borenstein, M. (2009). Effect sizes for continuous data. In H. Cooper, L. V. Hedges, & J. C. Valentine (Eds.), *The handbook of research synthesis and meta-analysis* (2nd ed.). New York, NY: Russell Sage Foundation.
